# New self-sexing *Aedes aegypti* strain eliminates barriers to scalable and sustainable vector control for governments and communities in dengue-prone environments

**DOI:** 10.3389/fbioe.2022.975786

**Published:** 2022-10-25

**Authors:** Siân A. M. Spinner, Zoe H. Barnes, Alin Mirel Puinean, Pam Gray, Tarig Dafa’alla, Caroline E. Phillips, Camila Nascimento de Souza, Tamires Fonseca Frazon, Kyla Ercit, Amandine Collado, Neil Naish, Edward Sulston, Gwilym C. Ll. Phillips, Kelleigh K. Greene, Mattia Poletto, Benjamin D. Sperry, Simon A. Warner, Nathan R. Rose, Grey K. Frandsen, Natalia C. Verza, Kevin J. Gorman, Kelly J. Matzen

**Affiliations:** ^1^ Oxitec Ltd., Abingdon, United Kingdom; ^2^ Oxitec do Brasil, Campinas, Brazil

**Keywords:** Aedes aegypti, vector control, self-limiting insects, genetic sexing, sustainability

## Abstract

For more than 60 years, efforts to develop mating-based mosquito control technologies have largely failed to produce solutions that are both effective and scalable, keeping them out of reach of most governments and communities in disease-impacted regions globally. High pest suppression levels in trials have yet to fully translate into broad and effective *Aedes aegypti* control solutions. Two primary challenges to date–the need for complex sex-sorting to prevent female releases, and cumbersome processes for rearing and releasing male adult mosquitoes–present significant barriers for existing methods. As the host range of *Aedes aegypti* continues to advance into new geographies due to increasing globalisation and climate change, traditional chemical-based approaches are under mounting pressure from both more stringent regulatory processes and the ongoing development of insecticide resistance. It is no exaggeration to state that new tools, which are equal parts effective and scalable, are needed now more than ever. This paper describes the development and field evaluation of a new self-sexing strain of *Aedes aegypti* that has been designed to combine targeted vector suppression, operational simplicity, and cost-effectiveness for use in disease-prone regions. This conditional, self-limiting trait uses the sex-determination gene *doublesex* linked to the tetracycline-off genetic switch to cause complete female lethality in early larval development. With no female progeny survival, sex sorting is no longer required, eliminating the need for large-scale mosquito production facilities or physical sex-separation. In deployment operations, this translates to the ability to generate multiple generations of suppression for each mosquito released, while being entirely self-limiting. To evaluate these potential benefits, a field trial was carried out in densely-populated urban, dengue-prone neighbourhoods in Brazil, wherein the strain was able to suppress wild mosquito populations by up to 96%, demonstrating the utility of this self-sexing approach for biological vector control. In doing so, it has shown that such strains offer the critical components necessary to make these tools highly accessible, and thus they harbour the potential to transition mating-based approaches to effective and sustainable vector control tools that are within reach of governments and at-risk communities who may have only limited resources.

## 1 Introduction

*Aedes aegypti*, the principal vector of dengue, chikungunya, Zika and yellow fever (Gubler, 2004; Jentes et al., 2011; Simmons et al., 2012; Weaver and Lecuit, 2015; Malone et al., 2016) is now widespread on every habitable continent of the world (Kraemer et al., 2015). Bites from *Aedes* mosquitoes cause millions of new human infections each year (Simmons et al., 2012), with mosquito control often being the only viable means of reducing disease incidence and spread (Ooi et al., 2006) as, with the exception of yellow fever, vaccines are commonly unavailable nor easily disseminated (Morrison et al., 2008). To enable a vector management technology to be used as an effective tool in the public health sector, where budgets are constrained and frequently only available as a reaction to new or sustained threats of disease, the ability to deploy widely, rapidly and economically is essential. This remains a difficult challenge to overcome and is one of the reasons for near-universal reliance on chemical insecticides for much of the last century. However, vector control using conventional insecticides is increasingly ineffective due to insecticide resistance (Vontas et al., 2012; Valle et al., 2019; Campos et al., 2020), and carries the risk of significant negative impacts on other species (Milam et al., 2000). Meanwhile, the prevalence of some *Aedes*-borne diseases such as dengue continues to rise rapidly (Messina et al., 2019).

An increasingly attractive vector management alternative is the use of biological, mating-based approaches to reduce populations of *Aedes aegypti*. These strategies employ male *Aedes aegypti* that when mated with wild females render some or all the offspring unviable. The sterile insect technique (SIT), involving irradiation of mosquitoes to induce sterility and physical sex-sorting to enable male-only releases, has historically not been successful for *Aedes aegypti* (Morlan et al., 1962; Ogah and Juma, 1977), though some recent successes in small-scale trials in Mexico (involving a combination of SIT and *Wolbachia*-infected males) and Cuba indicate a renewed interest in SIT for *Aedes aegypti* (Gato et al., 2021; Martín-Park et al., 2022).

The SIT has seen considerable successes for other insects such as the new world screwworm, the Mediterranean fruit fly, and other agricultural pests (Krafsur, 1998; Klassen and Curtis, 2005). Ongoing efforts to improve irradiation methods for *Aedes aegypti* may prove more successful in producing sterile males with sufficient fitness to mate effectively in the field (Bond et al., 2019). The development of incompatible insect techniques (IIT) using Wolbachia to induce cytoplasmic incompatibility (Mains et al., 2019; Crawford et al., 2020) and the development of genetically modified ‘self-limiting’ *Aedes aegypti* have both had significant successes in controlling this important vector and reducing its impact on local scales (Phuc et al., 2007; Harris et al., 2012; Carvalho et al., 2015; Gorman et al., 2016; Garziera et al., 2017).

Despite these technical demonstrations of performance, however, two major challenges to the deployment and scalability of these technologies remain: production of male-only cohorts, and handling and release of adult mosquitoes. The production of male-only cohorts of mosquitoes is important, as males do not bite nor transmit disease, and in some cases accidental female releases may undermine performance and reduce efficacy (Xi et al., 2005). The handling and release of adult mosquitoes is also a significant challenge. Following mass production in a dedicated facility and the associated quality control of batches, male-only cohorts must be dispatched for release by vehicle or by hand several times per week (Carvalho et al., 2015) or even daily (Crawford et al., 2020). Systems of this kind have recently been described for the production of *Wolbachia*-infected male *Aedes aegypti* (Crawford et al., 2020) and of irradiated, *Wolbachia*-infected male *Aedes albopictus* (Zheng et al., 2019). Both systems achieved effective population suppression in small local areas but each of the technologies requires localized, complex adult male mosquito rearing and releases, necessitating large numbers of staff, highly technical production systems, and offering only short timeframes within which to deploy. Improvements continue to be made in storage and release methods for male mosquitoes (Chung et al., 2018; Bouyer et al., 2020; Marina et al., 2022), but all current mosquito release approaches for vector suppression rely on labour-intensive and expensive sex-separation of mosquitoes prior to release.

For these reasons, and despite their clear performance potential, mating-based strategies remain limited in terms of scalability and broad accessibility. Due to the operational complexities, unavoidable biological constraints, and high associated costs, particularly in the context of developing countries where the need for mosquito control is often greatest, these approaches are likely to remain difficult to scale and thus potentially out of reach of communities most in need.

The development of a self-sexing (genetic) strain of *Aedes aegypti* has the potential to remove these limitations. Mosquito eggs of a genetic sexing strain will produce male-only cohorts of adult mosquitoes as all female larvae die during development. This eliminates the need for costly production facilities in release locations, as eggs can be produced in highly efficient facilities positioned centrally and stored and shipped globally. Egg-based deployment of the strain becomes possible, whereby eggs can be placed in mosquito rearing devices in the field, enabling male mosquitoes to develop and emerge naturally over an extended time period, thereby eliminating the production and adult release operations. By engineering the female lethality so it is repressible, the mass-production of mosquito eggs in a centralised colony is made possible, and logistics for the distribution of egg-based devices becomes efficient, practical, and low-cost.

In addition to removing the associated labour, equipment, and material costs, genetic sex-sorting has other significant advantages over manual sex-sorting: an improved safety profile as biting females cannot be unintentionally released; reduced handling of released insects, likely improving fitness and performance (Zheng et al., 2019; Crawford et al., 2020); and importantly the ability to deploy egg-based devices rapidly to a perceived threat in any location.

In this paper, the creation of a genetic sex-sorting, male-selecting *Aedes aegypti* strain with complete trait penetrance is described. This technology is different from self-sustaining gene-drive technologies designed to establish and spread within the environment (Sinkins and Gould, 2006) for which additional regulatory scrutiny may be a pre-requisite for field implementation (Devos et al., 2022). In contrast, the OX5034 gene is self-limiting and is engineered to disappear from the environment and will therefore neither establish nor spread (some gene drive approaches designed to limit environmental dispersal are also in development (Leftwich et al., 2018; Hay et al., 2021)). This male-selecting *Aedes aegypti* strain (known as OX5034 *Aedes aegypti*) was able to suppress wild *Aedes aegypti* populations in dense urban environments in Brazil, with peak suppression of up to 96% observed.

## 2 Materials and methods

### 2.1 Wild-type strain background

The Latin wild-type (WT) strain originates from Mexico and was field collected in Chiapas, Mexico in 2006 (Wise de Valdez et al., 2011) with the purpose of creating a “genetically diverse laboratory strain” (GDLS) which would maintain more genetic variation during laboratory colonisation than a single field collection (Wise de Valdez et al., 2010). Ten geographically separate field collections were performed around Chiapas and each collection was maintained as a separate colony. Individuals from these ten colonies were combined to create a single strain and transferred to Oxitec in 2006, which is now referred to as “Latin wild-type”. The strain is not reported to have any specific traits relevant to human health, i.e. increased or decreased vector competence, fecundity, *etc.*

### 2.2 Microinjection and isolation of strains

*Aedes aegypti* eggs of the Latin wild-type background (described above) were reared under standard insectary conditions: 26°C ± 2°C, 70% ± 10% relative humidity and 12 h: 12 h light: dark cycle. Larvae were fed ground Tetramin^®^ fish flakes and adults were provided with 10% sucrose solution. Eggs were obtained by providing mated females with defibrinated horse blood and allowing access to wet seed germination paper as an oviposition substrate.

Mosquito embryos were transformed by standard micro-injection methods as described by (Morris, 1997; Jasinskiene et al., 1998). Newly drawn needles were beveled using a microelectrode beveller (BV-10, Shutter Instrument, Co.) to optimize needle tip diameter. Injection was achieved using a combination of pOX5034 plasmid DNA (concentration of 300 ng/μl) and *piggyBac* mRNA (at a concentration of 500 ng/μl) as the source of transposase. The plasmid DNA and the transposase mRNA were reconstituted in an injection buffer (5 mM KCl, 0.1 mM NaH_2_PO_4_, pH 6.8) made using standard laboratory grade reagents (Handler and James, 1998). The transposase mRNA provides a source of *piggyBac* transposase, to allow the rDNA construct to be integrated into the germline of *Aedes aegypti*. The non-autonomous transposon has no endogenous source of transposase in mosquitoes and has had no further translocation.

pOX5034 was cloned from DNA fragments containing *piggyBac* 5ʹ and 3ʹ transposable elements, DsRed2, tTAV, nls, SV40 3ʹ UTR, and TetO x7, which were synthesised by GeneArt, while the *scraps* intron, ubiquitin and DmHsp70 minipromoter were previously amplified from *D. melanogaster* genomic DNA, and subcloned into pOX5034. Likewise, the *Aeadsx* splicing module was previously amplified from *Aedes aegypti* genomic DNA and subcloned into pOX5034, and the fragment of the *immediate-early1* (*ie1*) gene promoter with the HR5 enhancer (originally identified in *Autographa californica* nuclear polyhedrosis virus (AcMNPV) (Guarino and Summers, 1986; Guarino and Dong, 1991; Rodems and Friesen, 1993)) was previously amplified from AcMNPV genomic DNA and subcloned into pOX5034. All genetic components were assembled into a standard backbone vector (containing amp(R) and pUC ori) using standard restriction enzyme cloning and Gibson assembly-type methods (Gibson et al., 2009).

The female-specific self-limiting gene is conditional on the presence of tetracycline or suitable tetracycline analogues, such as doxycycline. OX5034 adult injection survivors (Generation 0 or G_0_) were back crossed to Latin WT and reared through larval life-stages in a rearing environment with doxycycline (‘on-dox’), deionized (dl) water containing 1 μg/ml doxycycline hydrochloride. Two G_0_ males were crossed to 10 Latin WT females and 6 G_0_ females were crossed with 6 Latin WT males. G_1_ pupae were screened for DsRed2 fluorescence using a Leica M80 microscope equipped with filters for detection: maximum excitation 563 nm, emission 582 nm. Three individual G_2_ males from transgenic families were crossed to WT females to create single insertion strains. Strains were maintained by crossing G_3_ males to Latin WT females.

**FIGURE 1:**
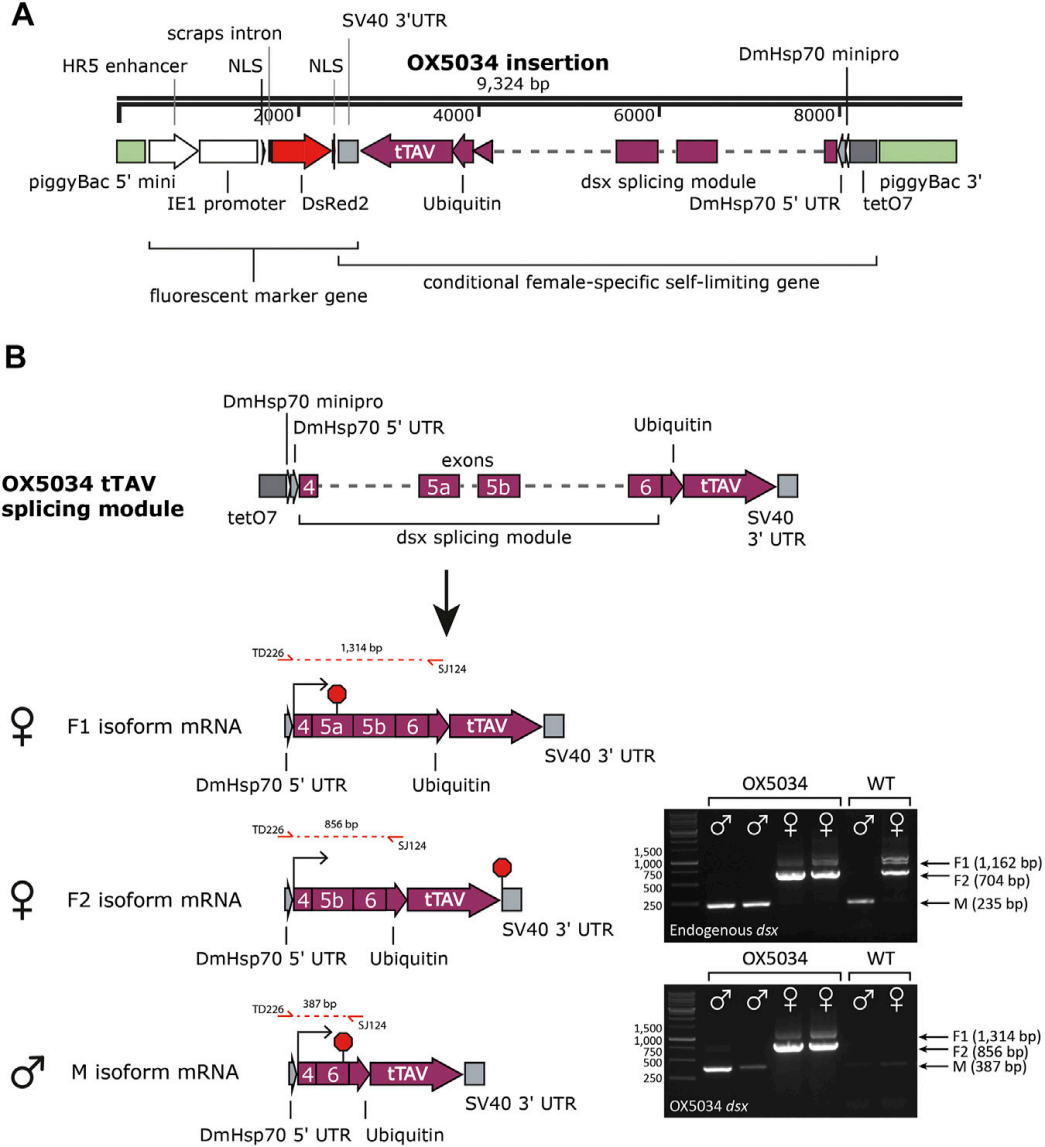
**(A)** Linear plasmid map showing the two genes (DsRed2 and tTAV) between the non-autonomous vector piggyBac sequences, inserted in the OX5034 *Aedes aegypti* strain. Due to the splicing module tTAV protein is only expressed in females in the absence of tetracycline family antibiotics. **(B)** Sex-specific splicing of endogenous *Aeadsx* and of the *Aeadsx*-ubiquitin-tTAV gene was confirmed in *Aedes aegypti* mosquitoes (pupal life stage, mosquitoes reared with doxycycline in the rearing medium) by RT-PCR reactions using either primers SS1997 and TD3349 (endogenous *dsx*), or TD226 and SJ124 (OX5034 *dsx*). All splicing isoforms of the endogenous *Aeadsx* gene (M, F1 and F2) were detected in WT as well as OX5034 mosquitoes in a sex-specific manner. The *Aeadsx*-ubiquitin-tTAV gene was also splicing in a sex-specific manner and was only detectable in mRNA extracted from OX5034 *Aedes aegypti* mosquitoes. In the absence of tetracycline antibiotics, the F2 isoform mRNA results in female-specific tTAV protein expression, and *dsx* and ubiquitin amino acids are cleaved from tTAV protein by endogenous cellular processes. Red octagons indicate stop codons.

Splicing of the *Aeadsx* gene in OX5034 was analysed by RT-PCR (Figure 1):

RNA was extracted from OX5034 and wild-type *Aedes aegypti* using the Total RNA Purification Plus Kit (Norgen), following the kit’s protocol for animal tissues. RNA was eluted in 20 μl. cDNA was synthesized from the RNA using the RevertAid First Strand cDNA Synthesis Kit (ThermoFisher). The resulting cDNA was used in the following PCRs: each reaction contained 10 μl PCRbio Taq Mix Red (PCRBiosystems); 1 μl each of 10 μM forward and reverse primer (see table); 3 μl cDNA and 5 μl pure water. Both endogenous and OX5034 construct splicing reactions used the same touchdown thermocycling conditions: 94°C for 2 min; (94°C for 15 s, 60°C for 30 s, decreasing by 0.5°C per cycle, 72°C for 15 s) x 10; (94°C for 15 s, 55°C for 30 s per cycle, 72°C for 15 s) x 25; 72°C for 7 min; hold at 12°C. Primers: TD3349 (Endogenous *Aeadsx* forward) TCA​ATG​GCT​CCT​GGA​GAA​GC; SS 1997 (Endogenous *Aeadsx* reverse) AAA​ATC​GGC​ATA​TGG​CGA​CCG​TGA​CGC​A; TD226 (OX5034 *Aeadsx* forward) AAG​TGA​ACA​CGT​CGC​TAA​GCG; SJ124 (OX5304 *Aeadsx* reverse) GGT​CAG​GGT​CTT​GAC​GAA​GAT.

### 2.3 Strain evaluation

To assess OX5034 *Aedes aegypti* homozygote, hemizygote and WT penetrance on and off doxycycline, four different crosses were set up as described in Table 1, i.e. homozygous OX5034 males crossed with homozygous OX5034 females, homozygous OX5034 males crossed with WT females, homozygous OX5034 females crossed with WT males, and WT males crossed with WT females. The homozygous OX5034 adult parents used in these crosses (male and female) were generated by rearing OX5034 with doxycycline (4 μg/ml) in larval diet, but not in adult sugar diet. For each cross, five cohorts (N = 5) of 200 L_1_ larvae (n = 200) were counted into new larval rearing containers and reared in 200 ml of deionised water without any tetracycline/doxycycline. An additional five cohorts (N = 5) of 200 L_1_ larvae (n = 200) were counted into rearing containers and reared in 200 ml of deionised water containing 4 μg/ml of doxycycline. All cohorts were reared and fed as described above.

**TABLE 1:** OX5034 *Aedes aegypti* homozygote, hemizygote and WT penetrance on and off doxycycline. Percentages are means of L_1_ individuals reaching the specified stage based on initial counts of 200 L_1_ larvae per repeat (5 repeats) and assuming a 1:1 sex ratio. 95% confidence intervals are displayed in parentheses. Note that in the case of female OX5034 progeny, zero female adults (functional or otherwise) were identified in any of the crosses without doxycycline.

Cross	Doxycycline concentration (µg/ml)	Male progeny		Female progeny	
		% Total Eclosion	% Functional Adults	% Total Eclosion	% Functional Adults
OX5034 ♂ x OX5034 ♀	0	69.8 (±8.8)	63.6 (±8.1)	(±0.0)	(±0.0)
	4	63.2 (±8.0)	58.8 (±9.1)	47.5 (±6.0)	43.0 (±6.8)
OX5034 ♂ x WT ♀	0	87.2 (±5.5)	61.4 (±5.5)	(±0.0)	(±0.0)
	4	87.7 (±5.5)	69.0 (±7.3)	75.3 (±5.9)	65.4 (±9.1)
OX5034 ♀ x WT ♂	0	70.6 (±11.2)	68.4 (±10.5)	(±0.0)	(±0.0)
	4	70.8 (±5.1)	68.0 (±5.9)	59.0 (±3.3)	57.8 (±3.6)
WT ♂ x WT ♀	0	65.0 (±3.9)	62.6 (±4.2)	60.3 (±5.4)	57.2 (±5.3)
	4	69.2 (±3.2)	67.6 (±3.3)	80.5 (±7.0)	76.8 (±7.1)

Upon pupation, dead larvae, dead pupae and live pupae were collected and counted daily. Pupae from each cohort were screened for DsRed2 fluorescence and males and females transferred to separate cages for eclosion and adult survival assessment. Adults were provided with a 10% sucrose solution. Three days after the last pupa was added, cage contents were counted and scored. The results shown in Table 1 are percentages as means of L_1_ individuals reaching the specified stage based on initial counts of 200 L_1_ larvae per repeat (5 repeats) and assuming a 1:1 sex ratio.

For longevity analysis, OX5034 males (homozygous and hemizygous) and females (homozygous only) were reared as described above, either with doxycycline (4 μg/ml) to generate female OX5034, or without antibiotics, to generate male OX5034. These adults were allowed to mate with either homozygous OX5034 or wild-type mosquitoes of the opposite sex for 2 days, prior to being isolated and left for longevity analysis. Four repeats (N = 4) of 25 adult males or 25 adult females (n = 25) were analysed. WT males and females were analysed in the same way for comparison. Adults were provided with 10% sucrose solution *ad libitum*, and female cages were provided with blood meals on days 7 and 17, with eggs collected on wet seed germination paper. Dead adults from all cages were removed daily and counted. Cages were rotated in the insectary to control for any environmental factors based on positioning in the insectary. For longevity data the RStudio Survival Analysis package, survival (2.38–3) was used to plot Kaplan-Meier curves and test for significance.

To measure male pupal size of OX5034 and WT males reared on and off doxycycline, between 100 and 150 male pupal cephalothorax dimensions were measured per rearing condition by imaging the dorsal view of pupae using a Canon IXUS 170 camera and cephalothorax width measured across its widest section using ImageJ 1.42q software. Data were analysed using Microsoft Excel version 16.0.6001.1038 and RStudio software package version 0.99.465 (RStudio, United States).

Male fitness analysis was carried out by rearing homozygous male OX5034 and WT mosquitoes as described above, with either 4 μg/ml doxycycline in the larval rearing medium, or without doxycycline (Table 2). Average survival to pupation from L_1_ was calculated from 5 replicate experiments (N = 5) each containing cohorts of 200 L_1_ larvae (n = 200).

**TABLE 2:** Comparison of average male pupal survival (assuming 1:1 sex ratio), peak pupation, cephalothorax width and adult longevity of OX5034 and WT strains reared under controlled laboratory conditions with and without 4 μg/ml doxycycline (±95% Confidence Intervals).

Doxycycline concentration	OX5034		Wild-type	
	4 μg/ml	0 μg/ml	4 μg/ml	0 μg/ml
MALES				
Pupal survival from L1	68.8% (±6.9)	74.2% (±8.6)	70.4% (±3.5)	66.8% (±3.5)
Age at peak pupation (days)	9 (% pupation on this day = 36.8% ± 2.27)	8 (% pupation on this day = 59.8% ± 8.93)	9 (% pupation on this day = 41.8% ± 7.58)	9 (% pupation on this day = 38.6 ± 3.20)
Average cephalothorax width (mm)	1.10 (±0.007)	1.01 (±0.009)	1.11 (±0.006)	1.09 (±0.007)
Median longevity (days)	39 (95% CI: 35–45 days)	24 (95% CI: 19–32 days)	39 (95% CI: 35–50 days)	49 (95% CI: 45–57 days)

To measure mating competitiveness, 15 replicate cages were set up, each containing 10 OX5034 males, 10 WT males, and 10 WT females. Following mating, females were removed, blood fed and transferred to individual Drosophila vials to lay eggs on damp cotton wool. Eggs were matured for 4–6 days and hatched. First instar larvae were screened for the presence of the fluorescent DsRed2 marker to determine paternity. Progeny were counted and the Relative Sterility Index (RSI) calculated according to the following equation:

$$RSI=\frac{TW}{\left(TW+WW\right)}$$

Where: TW = transgenic homozygous males (OX5034) mated with WT females, giving rise to fluorescent progeny. WW = WT males mated with WT females giving rise to non-fluorescent progeny.

And overall RSI is the average of the 15 replicate cages with 95% CI calculated.

In the instance of double mating, a point was awarded to both the transgenic male and WT male.

For the assessment of trait decline in caged populations, three cages (N = 3) containing 200 L_1_ larvae were set up. These L_1_ larvae (n = 200) were generated by crossing hemizygous male OX5034 with WT female *Aedes aegypti*, and thus represent a post-release field population with a male-selecting trait frequency of 0.25. Each of the three cohorts represent a population. Larvae were fed according to the standard feeding regimen. From day 7 post-hatching, pupae were collected, screened for fluorescence, sexed and counted. Sexes were kept in separate cages to allow eclosion and sexual maturation. Three days after the last pupa was added to the cage, males were transferred to the female cages for mating. Defibrinated horse blood was provided and F2 eggs collected on wet seed germination paper. This rearing process was repeated for subsequent generations, until the male-selecting trait was eliminated from each population based on the absence of fluorescent individuals in the population.

**FIGURE 2:**
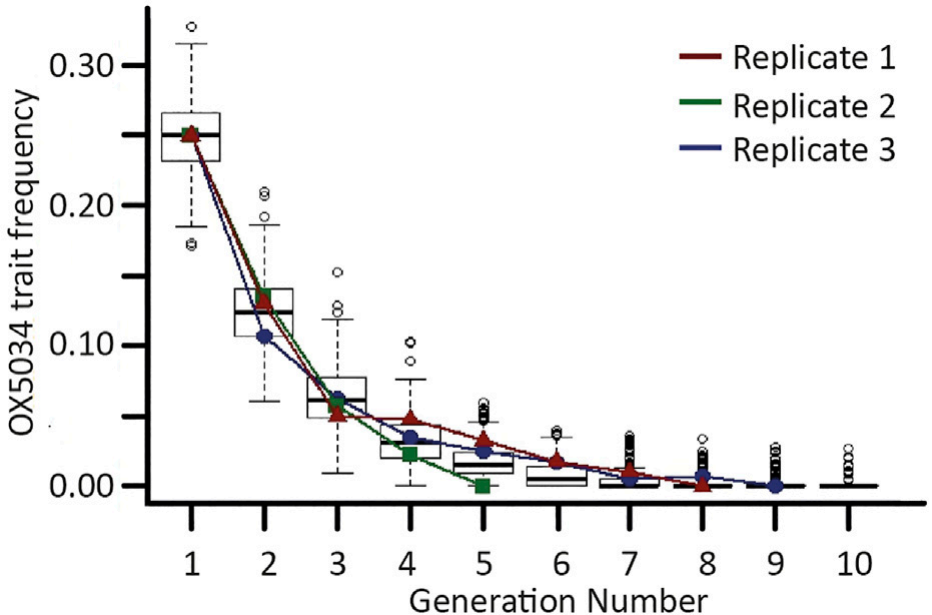
Trait decline of OX5034 *Aedes aegypti*. Boxplots showing the results from 500 iterations of a stochastic model simulating the extinction of a male-selecting genetic trait under restrictive conditions (off-doxycycline rearing). Horizontal bold lines represent generational medians; upper and lower box lines represent first and third quartiles, respectively; outer horizontal lines represent 1.5× the interquartile range; and open circles represent data points over 1.5× above or below the first and third quartiles. Overlaid onto the box plots are lines (red, blue and green) showing male-selecting trait frequency changes from three replicates of caged experiments. Generation 1 represents a post-field release population with a trait frequency of 0.25.

Results were compared to a stochastic model (Harvey-Samuel et al., 2014), designed to simulate male-selecting trait loss under conditions equivalent to our experimental populations (population size of 200, initial male-selecting trait frequency of 0.25, restrictive conditions). This model used Monte Carlo simulations to generate 500 independent populations, in each of which mating pairs and offspring were randomly selected from the population of the previous generation (Figure 2). The model assumed complete penetrance of transgenic females but no fitness penalty in transgenic males relative to WT males; corresponding to an overall 50% fitness penalty for the genetic trait. Therefore, the male-selecting trait frequency is predicted to halve in each generation. Data were analysed using Microsoft Excel version 16.0.7927.1020 and the RStudio software package, version 1.0.143 (RStudio, United states). Modelled and experimental data sets were compared using a 2-way repeated measure ANOVA.

### 2.4 OX5034 rearing for release

A colony of homozygous OX5034 established and maintained in Brazil under controlled environmental conditions (26–29°C; >70% RH; 12 h light cycle) was used to generate male mosquitoes for release.

Eggs were hatched in the absence of tetracycline/doxycycline and reared as larvae without tetracycline/doxycycline. Male pupae reared without doxycycline (to ensure male-only release cohorts) were aliquoted into ventilated plastic release pots, and water drained from pots once eclosion was complete. Six release pots per week were used for quality control (QC) purposes. Three of these QC pots were transported with the release batches and returned to the laboratory unopened, to assess the effects of transport on the released males. Three QC pots were kept in the lab. Flying male mosquitoes in all six QC pots were then counted and used to estimate release numbers accurately, and to verify that no female OX5034 mosquitoes were present in release cohorts.

### 2.5 OX5034 release methods

**FIGURE 3:**
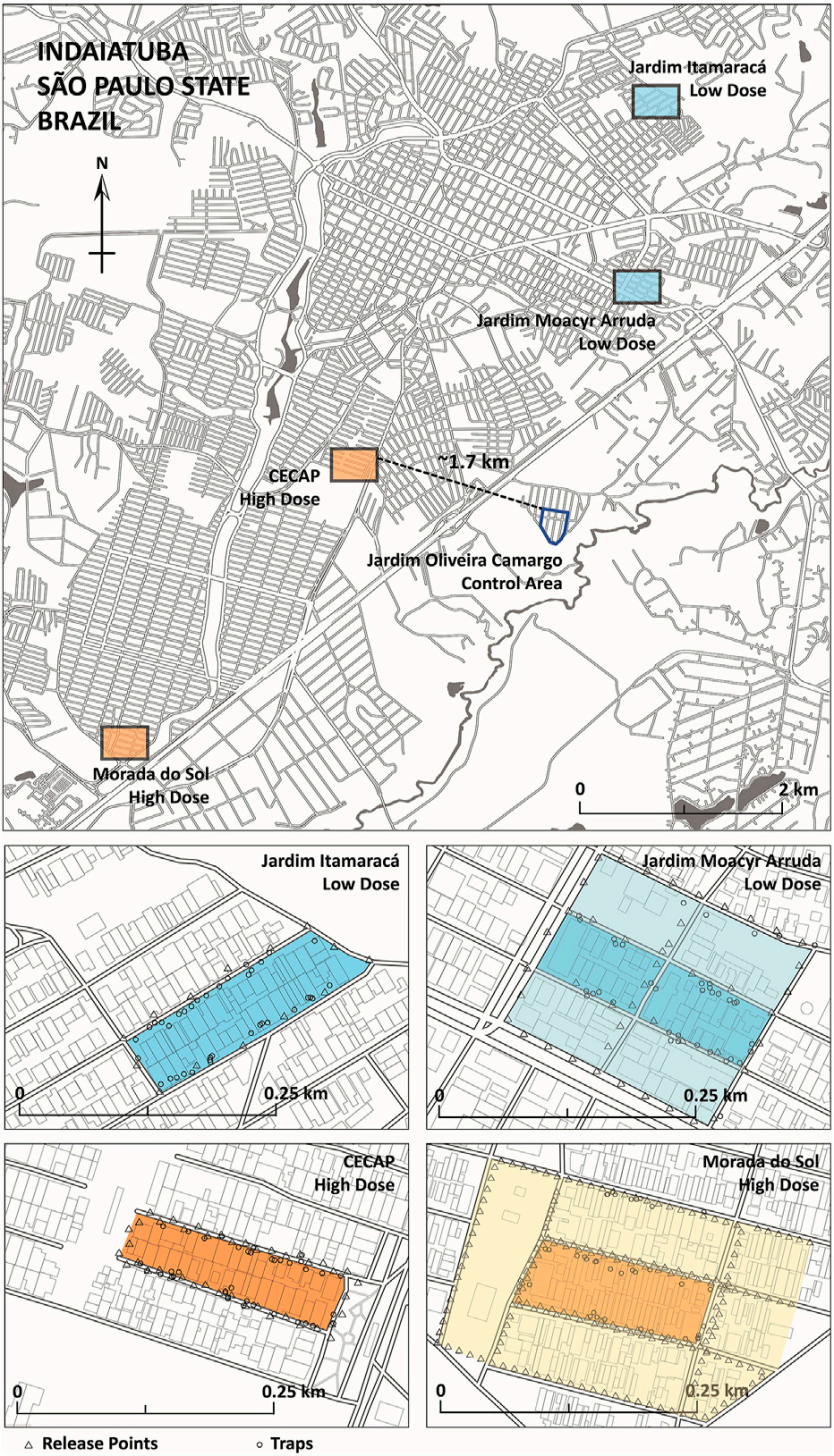
OX5034 *Aedes aegypti* release neighbourhoods in the city of Indaiatuba, São Paulo State. Four treatment areas are shown, with blue shading indicating low-dose treatment areas (Jardim Itamaracá and Jardim Moacyr Arruda), and orange shading indicating high-dose treatment areas (CECAP, Morada do Sol). The control area (Jardim Oliveira Camargo) is shown with a dark blue outline. The geographic coordinates of the city are 23°05′24″ S and 47°13′04″ W. Release points (triangles) and trap locations (small circles) are shown in each of the four treatment areas. Buffer zones (deployed from February 2019) are shown for Jardim Moacyr Arruda (pale blue shading) and Morada do Sol (pale orange shading).

The study was conducted within the limits of the urban area of Indaiatuba (Figure 3), in the state of São Paulo.

This field study was carried out under a permit from Brazil’s National Technical Commission on Biosafety, CTNBio, Permit Number Processo SEI no 01250.008745/2016–11.

The releases of OX5034 began during May 2018, continuing throughout the drier season (June-September 2018) and into the following wet season (October 2018-April 2019), when *Aedes aegypti* levels are typically high.

**FIGURE 4:**
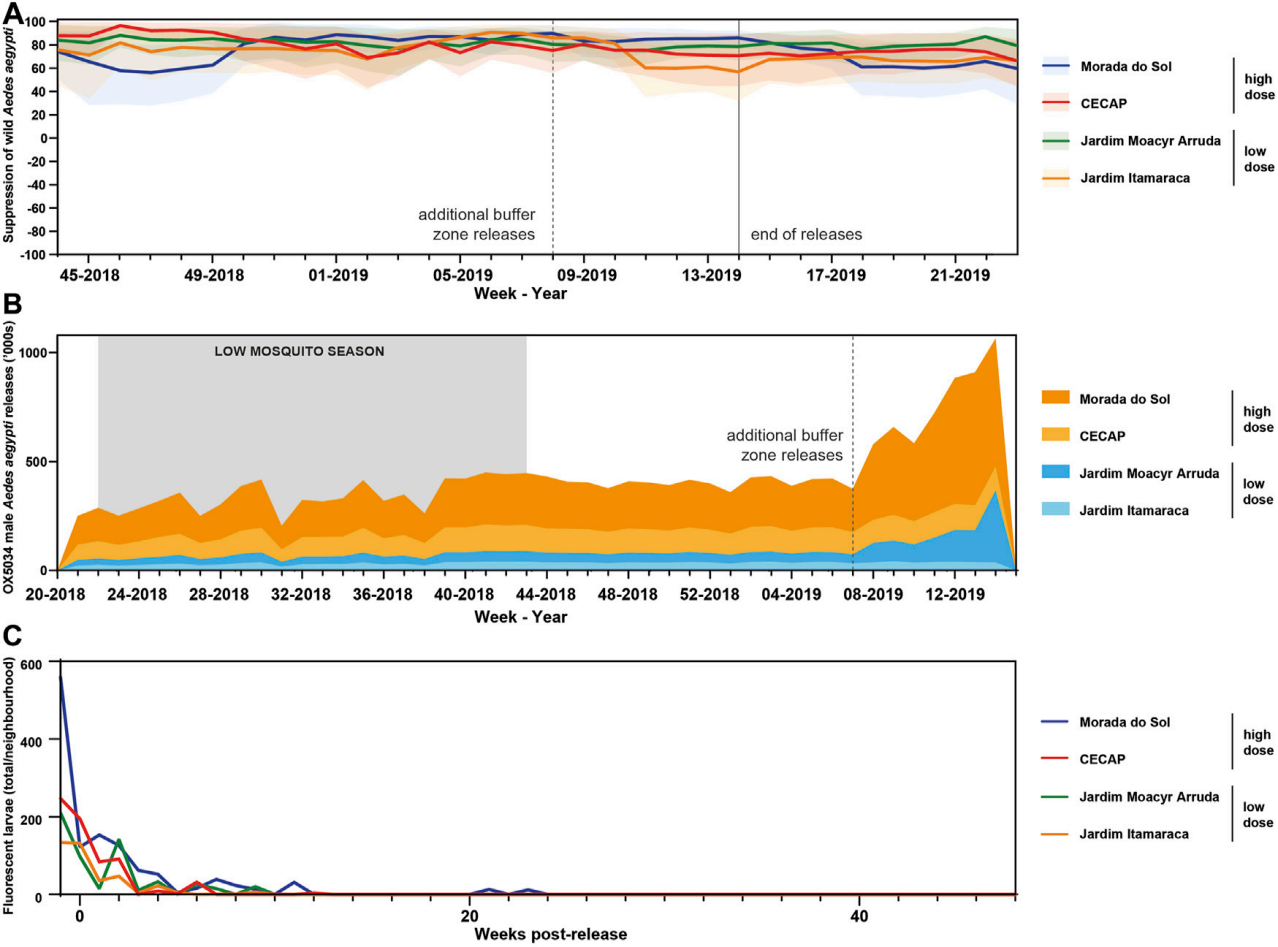
**(A)** Absolute suppression chart in all areas in Indaiatuba treated with OX5034 *Aedes aegypti* adult males. Solid lines indicate suppression calculated for each release area, relative to the untreated control area. Shaded areas indicate 95% confidence intervals. **(B)** OX5034 *Aedes aegypti* male mosquito releases during the Indaiatuba field trial. Releases began in May 2018 (Week 22) and ended in April 2019 (Week 14). Additional buffer zone releases were added to two neighbourhoods, Morada do Sol (high dose) and Jardim Moacyr Arruda (low dose) in February 2019. **(C)** Post-release monitoring of the four treatment neighbourhoods demonstrated that the transgenic mosquitoes were no longer detected in the environment after 13 weeks (CECAP, Jardim Itamaracá and Jardim Moacyr Arruda), and after 24 weeks in Morada do Sol, a high-dose area with added buffer zone. Monitoring continued until 48 weeks after the end of OX5034 *Aedes aegypti* male releases.

Although the chosen number of OX5034 males for release was targeted to control the local *Aedes aegypti* population, wild populations of *Aedes aegypti* are closely associated with human populations, and therefore the release rate (or dose rate) is described as “number of OX5034 males per person” (Gorman et al., 2016). Two target dose rates were chosen, a high rate of 500 OX5034 males per person per week, and a low rate of 100 OX5034 males per person per week (Figure 4).

To ensure a constant presence of OX5034 males, a treatment frequency of 3 times per week was used (Figure 4). Ventilated plastic pots containing male OX5034 mosquitoes were packed into temperature-controlled transport boxes and driven to the release site. Adult male mosquitoes were released by opening one plastic container (containing approximately 1,000 male OX5034) at each preselected release point, shaking gently so that the adult males were efficiently dispersed. After the release, the six pots previously identified for QC checks were assessed, and the numbers of insects remaining in each (dead and alive) were recorded. This release pattern was repeated 3 times per week (Monday, Wednesday and Friday) from May 2018 to April 2019.

### 2.6 OX5034 monitoring methods

Ovitraps are commonly used for monitoring *Aedes* mosquitoes, mimicking natural breeding sites in which females lay eggs (Reiter and Nathan, 2001; Hernandez-Avila et al., 2013). The ovitraps consisted of a pot containing water and a wooden paddle to act as a substrate on which females could lay eggs and were used to sample *Aedes aegypti* populations at the treated and untreated sites.

Following regulatory approvals and a period of engagement with Indaiatuba municipality and local communities, a network of ovitraps (approx. 30 per 1 ha site) was deployed in five neighbourhoods: CECAP, Morada do Sol, Jardim Itamaracá, Jardim Moacyr Arruda, and Jardim Oliveira Camargo (Table 3). *Aedes aegypti* abundance was measured in all study sites for 9 weeks prior to initiation of OX5034 male releases.

**TABLE 3:** Geographical coordinates of Treatment Areas and Control Areas, their approximate areas and estimated human population.

Location	Treatment	Latitude	Longitude	Approximate area (acres)	Estimated population
Jardim Itamaracá	OX5034	23°04′27,00″S	47°11′35,39″W	2.51	353
Jardim Moacyr Arruda	OX5034	23°05′29,81″S	47°11′41,65″W	2.56	431
CECAP	OX5034	23°06′26,46″S	47°13′12,35″W	2.32	222
Morada do Sol	OX5034	23°07′57,50″S	47°14′28,30″W	2.32	430
Jardim Oliveira Camargo	Control Area	23°06′48,17″S	47°12′08,08″W	2.40	452

Ovitraps were placed outside residential houses, in sheltered locations preferred by aedine species (e.g. under tables, in corners, near water storage). The written consent of each property owner/occupant was sought before placement. Between 31 and 35 ovitraps were used per treatment and control area. All ovitraps were examined and replaced weekly, returning the substrate, including any eggs, to the laboratory for inspection. Once at the laboratory, any eggs collected were air dried for a minimum of 5 days and hatched in sterilized tap water. At the first instar stage (L_1_), all hatched larvae were screened for the presence of the OX5034 fluorescence marker using a stereomicroscope (Leica MZ10F; Leica Microsystems AG, Wetzlar, Germany) at a wavelength of 561 nm, and numbers of fluorescent larvae were recorded. All fluorescent and non-fluorescent larvae were retained and reared to the third-instar stage (L_3_), at which point each individual was identified taxonomically to species level. Fluorescent larvae were allowed to develop to adulthood and the sex of each individual determined.

BG-Sentinel^®^ traps were used to trap adult mosquitoes. These traps use both olfactory and visual cues to attract mosquitoes. In a similar manner to ovitraps, between two and six BG-Sentinel^®^ traps per site were deployed externally, near domestic houses at locations considered to be attractive to *Aedes aegypti*, and the written consent of each property owner/occupant was sought before placement. These were examined and replaced weekly (except for mark-release-recapture studies, where they were examined daily), returning the catches to the laboratory for inspection. Adult mosquitoes caught in BG traps were identified to species level and counted, and their sex was determined using a stereomicroscope. Adult trap data contributed to population estimates for OX5034 and native *Aedes aegypti*.

### 2.7 Mark-release-recapture methods

Approximately 1000 OX5034 male pupae were placed in a clear plastic pot coated with a fluorescent dye (DayGlo Color Corp, United states). Thirty-four pots were prepared in this way and stored at 26 ± 1°C and 60–70% relative humidity. Adults were allowed to eclose in the lab, and the hatched adult mosquitoes were fed *ad libitum* with 10% sugar solution and matured for 2–4 days before release. Three powder colours - Pink (Ax21), Yellow (Ax17) and Blue (A19) were used to mark different batches of mosquitoes which were released on successive days to provide replication for the study. The study was repeated once more 20 days after the initiation of the first MRR study, resulting in 6 independent replicates.

Quality control was performed on four of the prepared pots to determine the male emergence and functionality (ability to fly) while the rest (thirty pots) were transported under triple containment in air-conditioned vans following standard biosafety procedures. The mosquitoes were released from a single location in the middle of a BG Sentinel^®^ network in Jardim Itamaracá, Indaiatuba.

Following the release of the marked mosquitoes, the traps were monitored daily until 3 consecutive days with no powder-marked mosquitoes. The daily catch was contained and labelled following biosafety regulations and returned to the laboratory, where it was frozen at −20°C for a minimum of 1 h. The mosquitoes from each bag were then sorted, identified, and the powder-marked mosquitoes counted.

### 2.8 Statistical methods

Statistical analysis was were performed using R version 4.0.0. (R Core Team, 2018). From the spatial and temporal distribution of trapped mosquitoes, the dispersal was analysed using the Mean Distance Travelled (MDT) method which provides corrections for trap density (Morris et al., 1991) and the survival in the field was estimated using Buonaccorsi’s non-linear method (Buonaccorsi et al., 2003). Life expectancy was derived from the survival estimate using the formula -1/log_e_ (survival) (Niebylski and Craig, 1994).

Unless otherwise stated, confidence intervals (CIs) are reported at the 95% level, and data are presented to two significant figures. Significant differences were classified by non-overlapping 95% confidence intervals. For abundance of *Aedes aegypti*, the following formula was used:

$$O_{A}=\frac{A}{T}$$

Where *O*_*A*_ = average of WT *Aedes aegypti* larvae per trap. *A* = total number of WT *Aedes aegypti* larvae collected in all ovitraps. *T* = total number of recovered ovitraps.

Suppression was defined by use of 4-week rolling averages (i.e. simple, unweighted averages of the abundance over the 4-week period prior to the date) relative to the same period at each untreated site (Gorman et al., 2016), which were calculated according to the equation:

$$Sa\;\left(\%\right)=\left(1-\frac{Ta}{Ua}\right)\times 100$$

Where *Sa* = Absolute suppression. *Ta* = Mosquito abundance in the Treated area. *Ua* = Mosquito abundance in the Untreated (Control) area.

The corresponding 95% CIs were calculated by a 10,000-loop bootstrap for each period (Manly, 2006).

## 3 Results

### 3.1 Development of a genetic sexing strain of *Aedes aegypti*, OX5034

The sexing of insects using genetic means is well-documented (Thomas et al., 2000), and precision-engineered genetic sexing mechanisms have been successfully used in Mediterranean fruit fly (*Ceratitis capitata*) (Ogaugwu et al., 2013; Leftwich et al., 2014), diamondback moth (*Plutella xylostella*) (Jin et al., 2013), Caribbean fruit fly (*Anastrepha suspensa*) (Schetelig and Handler, 2012), olive fly (*Bactrocera oleae*) (Ant et al., 2012), *Drosophila suzukii* (Li et al., 2021), and the livestock pests sheep blowfly (*Lucilia cuprina*) and New World screwworm (*Cochliomyia hominivorax*) (Concha et al., 2020; Yan and Scott, 2020). In most of these cases, female-specific expression of the tetracycline-repressible lethal gene was achieved by linking the tTAV gene to a sex-specific splicing module derived from a gene involved in the sex-determination pathway. Hence in the absence of tetracycline, only males develop. The tTAV gene is a tTA variant sequence optimised for expression in *Drosophila melanogaster* and other insects, and forms part of the widely-used tet-repressible control system (Gossen and Bujard, 1992). High level expression of tTAV is thought to be deleterious to cells, likely due to transcriptional “squelching” (Gill and Ptashne, 1988). tTAV protein binds to and activates expression from the tetracycline response element (TRE) which includes the specific DNA sequence to which tTAV binds (tetO). tTAV also binds tetracycline with a high affinity, preventing it from binding DNA. tTAV acts, therefore as a tetracycline-regulated switch. High levels of tTAV expression is deleterious to cells as it can repress normal transcription. tTAV has been used in fungi, mice, plants and mammalian cultures with no known adverse effects on the environment or human health.

Many dipterans, including *Drosophila melanogaster* and the mosquito *Anopheles gambiae* have heteromorphic (XY) sex chromosomes, but sex chromosomes of *Aedes* mosquitoes are homomorphic in both sexes (Gilchrist and Haldane, 1946; Charlesworth and Mank, 2010). Sex is instead determined by the presence or absence of a dominant M-factor which is responsible for initiating male development (McClelland, 1962; Hall et al., 2015; Turner et al., 2018). This M-factor initiates a cascade of genetic mechanisms that culminates in the differential splicing of the doublesex (*dsx*) and fruitless genes between males and females, which then regulate an array of genes that determine sex-specific morphology and behaviour. We exploited the sex-specific differential splicing mechanisms from an abbreviated version of *dsx* by linking it to the tetracycline-off system to create a genetic sexing strain of *Aedes aegypti*, known as OX5034. (Sex-specific alternative splicing has also been adopted in *An. gambiae* to produce a male-specific EGFP reporter strain of *An. gambiae* (Magnusson et al., 2011)).

The endogenous *Aedes aegypti* doublesex (*Aeadsx;* GI 5571521) gene is large (450 kb) and consists of eight exons and seven introns (Salvemini et al., 2011). Only exons 4, 5a, 5b and 6, and introns 4, 5 and 6 are differentially spliced between males and females, and so these exons and introns were used to achieve sex-specific expression of the tTAV gene in OX5034. The sequence of some of these components (introns 4, intron 6 and exon 5b) was manipulated before being integrated in the sex-specific splicing module. Introns 4 and 6 were shortened (to 1,175 bp for intron 4 and 1,446 bp for intron 6) by retaining the 5′ and 3’ ends of each intron. Further insertions, deletions, and substitutions were made to exons 5b and 6, to create an open reading frame that encodes tTAV protein only in female isoform F2 (Figure 1 and Supporting Information Supplementary Table S1). As the *Aeadsx* sequences will lead to additional amino acids included on the N-terminus of the tTAV protein, the ubiquitin protein sequence (Ubi) was placed between the *Aeadsx* and tTAV sequences. Ubiquitin is cleaved through normal cellular processes, and so the *Aeadsx*-derived and ubiquitin amino acids are removed, producing unmodified tTAV (Bachmair et al., 1986; Varshavsky, 2005). When tetracycline is absent, tTAV binds to tetO and drives high levels of tTAV expression in females in a positive feedback loop that leads to cell death. When tetracycline is present, tTAV preferentially binds to tetracycline instead of tetO, and the insect cells function as normal (Gossen and Bujard, 1992; Phuc et al., 2007).

The OX5034 construct, which consists of the tTAV gene linked to *dsx*, together with a somatic fluorescent marker gene (Figure 1), was injected into 2,465 wild-type (WT) *Aedes aegypti* embryos, from a strain originally collected in Chiapas, Mexico (Wise de Valdez et al., 2011). Integration into the *Aedes aegypti* genome was achieved using the piggyBac non-autonomous transposase. Fluorescent scoring of G_1_ progeny identified 10 transgenic families (8 from male G_0_ crosses, 2 from female G_0_ crosses) from which 3 individual G_2_ males were crossed to WT females to create single insertion strains. Viable eggs were produced from 20 transgenic strains. Based on sex ratios of fluorescent and non-fluorescent pupae, 15 of the OX5034 strains displayed the desired male-selecting phenotype when reared under conditions without tetracyclines in the diet, with female rescue observed under rearing conditions where tetracyclines were added to the rearing diet. Initial assessment of the transgene-homozygous viability of each strain was carried out by screening the pupae from hemizygous crosses according to Mendelian genetics for expression of the DsRed2 marker (3:1 ratio fluorescent: non fluorescent). Only two strains demonstrated acceptable survivorship of homozygous transgenic individuals of both sexes in the presence of tetracyclines. Of these, based on sex ratios of fluorescent and non-fluorescent pupae, only one OX5034 transgenic family displayed the desired male-selecting phenotype penetrance, i.e. complete female death when reared in the absence of doxycycline, with female rescue in the presence of doxycycline. The absence of late-instar dead larvae or female pupae in off-doxycycline cohorts indicated female-specific tTAV expression was occurring in early larval stages. After crossing hemizygous OX5034 parents, the observed fluorescent to WT ratios of offspring (N = 3, n = 400 L_1_ larvae) were not significantly different from those predicted by Mendelian genetics (χ2 = 0.25, *p* = 0.62), indicating that there is no significant mortality caused by carrying two copies of the transgene. A homozygous strain of OX5034 *Aedes aegypti* (hereafter referred to as “OX5034”) was produced from crosses of 16 founding parents.

OX5034 rDNA was found to be inserted in the intronic sequence of the predicted *Aedes aegypti* gene AAEL009706, likely located on chromosome 3 (Matthews et al., 2018). The insertion site lies in intron 1 (30.5 kb in size), 1.9 kb upstream of exon 2 of AAEL009706, which codes for an unknown protein. Inside the same intron of this gene is another predicted gene (AAEL018077/AAEL009696), also coding for an unknown protein, which lies 9.1 kb upstream of the OX5034 rDNA site of insertion. Thus, the OX5034 rDNA insertion does not appear to disrupt any known *Aedes aegypti* protein-coding sequences. Sex-specific splicing of the *dsx*-tTAV gene was confirmed by RT-PCR (Figure 1).

### 3.2 OX5034 strain evaluation

**FIGURE 5:**
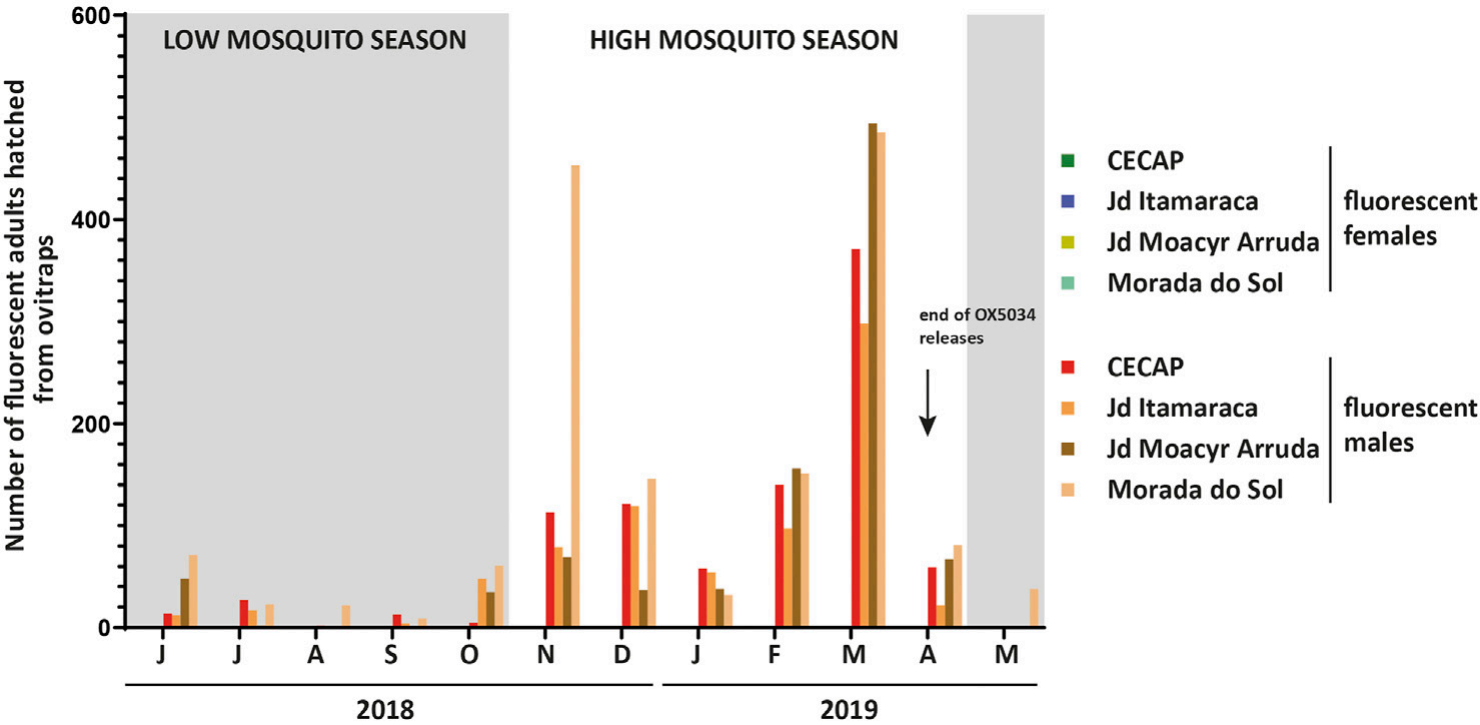
Number of fluorescent adults hatched from ovitraps-collected eggs from OX5034-treated areas, Indaiatuba, São Paulo State, Brazil. Each bar represents fluorescent adults from one of the four field sites (CECAP, Jardim Itamaracá, Jardim Moacyr Arruda, Morada do Sol). Low mosquito seasons (May-October) are shown with grey boxes. No fluorescent females were obtained from any ovitrap-collected eggs at any time throughout the 2018–2019 field trial of OX5034 *Aedes aegypti*. Data are reported as totals for each calendar month throughout the OX5034 *Aedes aegypti* release period.

The penetrance of the conditional female-specific self-limiting gene, i.e. the proportion of female insects that die before reproductive age, was assessed in OX5034. Both homozygous and hemizygous OX5034 individuals showed 100% female penetrance in the sample sizes tested in the laboratory and in field deployments (see Figure 5), with no adult female progeny detected when reared in the absence of doxycycline (see Table 1). This confirmed that one copy of the transgene was sufficient to kill the females, with this effect being dependent on the absence of doxycycline or other tetracycline antibiotics.

Homozygous and hemizygous males appear unaffected by the transgene (see Table 1), with the proportion of functional (i.e. flying) males proving not significantly different from that of WT (Wilcoxon test, homozygotes, w = 6.5, *p* = 0.25; hemizygotes (male homozygote OX5034 cross to WT), t = 0.039, df (degrees of freedom) = 5.48, *p* = 0.71; hemizygotes (female homozygote OX5034 cross to WT), two sample *t*-test, equal variance t = 0.14, df = 6.34, *p* = 0.89; N = 5, n = 200 for all comparisons).

Rescue of females carrying the transgene occurs when larvae are reared in the presence of doxycycline, albeit with some fitness penalties to females, with a statistically significant difference observed between WT females and OX5034 homozygous females (t = 6.34, df = 7.67; *p*-value = 0.0003; N = 5, n = 200 for all comparisons), with only 43% female rescue observed in the latter group. Hemizygous female progeny from male homozygote crosses to WT, and hemizygous progeny of female homozygote crosses to WT, also show decreased rescue compared to WT (65.4% (t = 1.92, df = 7.87, *p* = 0.09) and 57.8% (t = 4.38, df = 5.41, *p* = 0.006) rescue respectively compared to 76.8% for WT; N = 5, n = 200 for all comparisons) (see Table 1), indicating a modest fitness penalty when carrying the transgene, possibly due to slight leakiness of the tTAV system in females. Individuals carrying two copies of the transgene show reduced survival (i.e. larval death) compared to individuals carrying a single copy, as seen by 14.8% more hemizygotes becoming functional (i.e. flying) females compared to homozygotes (t = 3.71, df = 6.07, *p* = 0.01; N = 5, n = 200 for all comparisons).

OX5034 was also compared with the parental, unmodified WT strain, in terms of developmental rates from egg to adult, adult longevity and male mating competitiveness, when reared under controlled laboratory conditions. The proportion of OX5034 male larvae surviving to pupation off-doxycycline was significantly higher than WT (OX5034 74.2%, WT 66.8%, χ2 = 6.6, *p* = 0.01, N = 5, n = 200), and OX5034 peak male pupation occurred 1 day earlier than in WT mosquitoes (Table 2). OX5034 and WT larvae reared on doxycycline produced larger pupae on average (mean cephalothorax width OX5034 = 1.11 mm ± 0.007 mm, WT = 1.11 mm ± 0.006 mm, respectively) than their counterparts reared off-doxycycline (mean cephalothorax width OX5034 = 1.01 mm ± 0.009 mm, WT = 1.09 mm ± 0.007 mm). No significant size difference was found between OX5034 males (n = 137) and WT males (n = 145) reared on-doxycycline (Welch two-sample *t*-test t = 1.65, df = 272.4, *p* = 0.10). OX5034 male pupae (n = 119) reared off-doxycycline were significantly smaller than WT pupae reared off doxycycline (n = 133) (Welch two-sample *t*-test t = 12.36, df = 226.5, *p* < 0.0001) (Table 2).

Homozygous OX5034 males reared off-doxycycline have a reduced median longevity compared to their WT counterparts (OX5034 24 days, WT 49 days, log-rank χ2 = 51.2, *p* < 0.0001, N = 4, n = 25). On doxycycline, there is no significant difference in median longevity between homozygous OX5034 males and WT males (OX5034 39 days, WT 39 days, log rank χ2 = 1.9, *p* = 0.164, N = 4, n = 25) (Table 2). No significant difference was observed between the survival rates of hemizygous OX5034 males and their WT male counterparts when reared off-doxycycline (χ2 = 0.1, *p* = 0.712, N = 4, n = 25). The median survival was 44 days for OX5034 hemizygous males and 50 days for WT males. Homozygous OX5034 females reared on-doxycycline (4 μg/ml) have a reduced survivability compared to their WT counterparts (χ2 = 18.3, *p* < 0.0001, N = 4, n = 25). Their median survival is 42 days and 56 days, respectively.

Mating competitiveness of OX5034 homozygous males was compared to WT males competing for WT females (in a ratio of 1:1:1, respectively) in a cage. The relative sterility index (RSI) of OX5034 males was slightly lower, but not significantly different to that of WT males (OX5034 RSI = 0.44 (95% CI: 0.373–0.525), WT RSI = 0.56, χ2 = 1.98, *p* = 0.16).

OX5034 was also shown to be completely susceptible at WHO-recommended discriminating doses to commonly used adulticides for mosquito control, including deltamethrin, permethrin, malathion and the larvicide temephos (Supplemantary Datasheet S1).

### 3.3 OX5034 completely disappears from the environment over time

To demonstrate that the OX5034 self-limiting gene does not persist or spread in a wild receiving population, caged experiments were conducted to show the extinction of the genetic trait from a receiving population. The introduction of the OX5034 male-selecting trait into caged, WT *Aedes aegypti* populations and the subsequent off-doxycycline rearing of following generations was designed to replicate the termination of male-only releases.

The extinction rate of the OX5034 male-selecting trait in three separately caged populations was statistically consistent with modelled data (f-value = 0.0006, *p*-value = 0.981), with the male-selecting trait being lost from each experimental population within 10 generations. Given an initial male-selecting trait frequency of 0.25 in a closed population of 200 individuals, under restrictive conditions, the rate of loss of the genetic trait fell within the variation predicted by this model (Figure 2). The mean number of generations until disappearance of the OX5034 male-selecting trait was 7.3 (±1.2 SE) with the maximum number of generations until extinction of this trait being 9 (blue line). The model predicted that the male-selecting trait frequency would halve in each generation due to the 50% fitness penalty of the genetic trait (i.e. complete penetrance of transgenic females but no fitness penalty in transgenic males relative to WT males). This was observed in the experimental cages, with the average frequency of the male-selecting trait decreasing in each generation by 54% (±7.0 SE).

These data were also consistent with previous laboratory studies on Oxitec’s male-selecting strains of diamondback moth and olive fly, where the mean number of generations until extinction of the self-limiting trait was 6.0 (±0.58 SE) and 8.0 (±1.16 SE) respectively, with the respective maximum number of generations until extinction of the genetic trait being 7 and 11 (Harvey-Samuel et al., 2014). These results demonstrate the important difference between the use of a self-limiting male-selecting strain for population control, and the use of gene drive strains which are designed to persist and spread in a wild population. In real-world applications, this difference affords multi-generational suppression benefits to vector control programs without the need to consider factors relevant to irreversible methods.

### 3.4 Design of the field performance experiments for the OX5034 strain

The field performance of the OX5034 genetic sexing strain of *Aedes aegypti* was evaluated in an open field release trial in Indaiatuba, São Paulo State, Brazil. Releases of OX5034 adult male mosquitoes were conducted over an 11-month period in four city neighbourhoods in Indaiatuba, between May 2018 and April 2019. The study was conducted within the limits of the urban area of Indaiatuba (Figure 1), in the state of São Paulo. Following regulatory approvals and a period of engagement with Indaiatuba municipality and local communities, a network of ovitraps was deployed in four Treatment neighbourhoods and one untreated Control neighbourhood (Table 3). *Aedes aegypti* abundance was measured in all study sites for 9 weeks prior to initiation of OX5034 male releases.

OX5034 male pupae, produced using Oxitec standard operating procedures, were aliquoted into custom-designed release pots and kept at 26 ± 1°C, 60–70% relative humidity until emergence. Prior to the transport to release sites, 6 pots were randomly identified for quality control, and the functional mosquitoes counted before and after the transport to the release site, to determine the impact of the transport conditions. These numbers were used to estimate the weekly release rate to ensure the weekly target was achieved. During this time, the mosquito population in both Control and Treated areas was monitored weekly using a network of ovitraps and BG-Sentinel^®^ traps (Biogents, Regensburg, Germany).

### 3.5 Dispersal and longevity of OX5034 male mosquitoes

The longevity and dispersal of OX5034 adult male mosquitoes were determined using the Mark-Release-Recapture (MRR) method (Supplemantary Datasheet S1). In brief, the study consisted of marking the OX5034 adult males with a non-toxic coloured powder and releasing them from a fixed location in the middle of a BG Sentinel^®^ adult-mosquito trap network. The traps were collected daily and the coloured mosquitoes identified and counted in the laboratory.

Under these field conditions, the maximum observed distance travelled by the OX5034 adult males was 198 m and the mean distance travelled was 54.8 m. Released male mosquitoes survived for a maximum of 7 days with a mean longevity of 1.3 days. The mean longevity was comparable with previous field measurements for transgenic mosquitoes (Lacroix et al., 2012), yet lower than a value of 2.3 days published for WT *Aedes aegypti* in a study conducted in Panama (Neira et al., 2014).

### 3.6 Suppression of the wild mosquito population by OX5034 male mosquito releases

Suppression of wild *Aedes aegypti* populations was measured over the course of the 11-month field trial of OX5034 (Supplemantary Datasheet S1). Following release, male OX5034 mate with the wild *Ae. aegypti* females and all progeny inherit the self-limiting gene. While the male progeny will develop into adults and have the potential to pass on the self-limiting gene to subsequent generations of mosquitoes, all female offspring will die before reaching adulthood, leading to a reduction in the size of the wild population.

In this study, two different doses of adult male mosquitoes were released to compare the effectiveness—100 mosquitoes per person per week (Jardim Itamaracá and Jardim Moacyr Arruda) and 500 mosquitoes per person per week (CECAP and Morada do Sol). In addition, the releases in two of the treated areas (Jardim Moacyr Arruda and Morada do Sol) were extended to include buffer zones in February 2019, to investigate whether wild female mosquitoes immigrating into the treatment areas might impact the ability of OX5034 males to suppress the wild mosquito population (Figure 1). All sites were chosen to be as similar as possible, and all are densely-populated urban areas in Indaiatuba, São Paulo State, Brazil.

Eggs collected weekly from ovitraps in treated and untreated areas were hatched in the lab and reared to adulthood for sex and species identifications. Mosquito abundance was defined based on ovitrap monitoring of *Aedes aegypti*.

During the peak mosquito season (November 2018–April 2019) maximum suppression values calculated using a 4-week rolling average varied between 88% and 96%, whilst mean values for each site across the entire high season ranged between 72% and 81% (Table 4). Plotting suppression using 4-week rolling averages ensures single data points are not disproportionately represented (Gorman et al., 2016), and Figure 5 shows that consistently high suppression values were sustained in all four treated sites. Despite the small size of the neighbourhoods treated, which might be subject to the immigration of wild female mosquitoes, OX5034 males were effective in suppressing the wild population in all four treatment areas across the whole of the 2018–2019 mosquito season, even without the addition of buffer zones, indicating that OX5034 is a highly effective tool to suppress wild *Aedes aegypti* even in small areas with high mosquito pressure.

**TABLE 4:** The maximum and average suppression values calculated across the high mosquito season in the four treated areas in Indaiatuba. Maximum 4-week suppression is highest suppression observed when measured as a 4-week rolling average, with 95% confidence intervals calculated using the bootstrap method described above. The average suppression across the whole season is calculated as the mean of all 4-week rolling averages, across the high mosquito season.

Treatment	Dose (mosquitoes/person/week)	Buffer zone from February 2019	Maximum 4-week suppression (high season) (95%CI)	Average 4-week suppression (high season)
CECAP	500	No	96% (92%–100%)	78%
Morada do Sol	500	Yes	90% (81%–97%)	81%
Jardim Itamaracá	100	No	91% (82%–97%)	72%
Jardim Moacyr Arruda	100	Yes	88% (78%–96%)	81%

After releases ended, the persistence of the OX5034 genes in the wild population was assessed by continued ovitrap monitoring (Figure 5C). From 13 weeks following the cessation of releases, no OX5034 fluorescent progeny were detected in three out of four release areas. The fourth release area, Morada do Sol, had no detectable OX5034 fluorescent progeny from 24 weeks of the end of OX5034 male releases.

### 3.7 Performance of the OX5034 self-limiting gene in field populations

Trait penetrance was also assessed in field-collected samples. Eggs from ovitraps were matured and hatched, and all larval progeny were reared in the absence of tetracycline antibiotics to allow the assessment of field penetrance of the OX5034 strain compared against the local Brazilian wild *Aedes aegypti*. The species of every larva was identified, and the sex of each *Aedes aegypti* mosquito was recorded after eclosion into adulthood.

79,559 larvae were hatched from the control and four treatment areas. Of these, 12,902 were fluorescent when screened at the L_1_ larval instar stage. 4,193 of these fluorescent larvae survived lab rearing (in the absence of tetracyclines) and eclosed into adults. At least half of the 12,902 fluorescent L_1_ larvae were expected to die because of the female-specific self-limiting gene, based on a hypothetical male:female sex ratio of 1:1. Some degree of further losses are also to be expected during lab rearing of wild-caught mosquitoes. Fluorescent adult mosquitoes were sexed, and the numbers of males and females recorded (Figure 3).

No fluorescent females were seen in any of the lab-reared mosquito cohorts obtained from the field-collected eggs. In contrast, the survival rate of WT females to pupation/adulthood (calculated in Jardim Oliveira Camargo from the L_4_ larval stage, following species separation from *Aedes albopictus*) was 52 ± 9% (mean ±95% CI, 62 weeks of ovitrap monitoring data), indicating that the absence of fluorescent female adults could be attributed only to the action of the OX5034 self-limiting gene. This demonstrated that the OX5034 conditional female-specific self-limiting trait, conferred by the tTAV-OX5034 gene, was 100% penetrant against wild *Aedes aegypti* from this location, thereby providing 100% female-specific larvicidal efficacy.

## 4 Discussion

This paper describes the development and first field releases of an effective genetic sexing strain of *Aedes aegypti*, which has the potential to dramatically improve access to biological control of this important disease vector; this has been long sought-after (Gilles et al., 2014) in the field of mating-based vector control technologies. The OX5034 strain efficiently reduced abundance of wild *Aedes aegypti* by up to 96% in these disease-endemic neighbourhoods with high mosquito pressures, employing OX5034 male mosquito doses within the range of those that had been successful in other studies with a predecessor self-limiting *Ae. aegypti* strain (Carvalho et al., 2015; Gorman et al., 2016). However, it is conceivable that even lower doses than those assessed would still have been effective. The male-selecting self-limiting gene ensured no surviving female progeny from OX5034 male mosquitoes; 100% of the female progeny tested died before reaching adulthood demonstrating complete penetrance of the self-limiting trait in small-scale laboratory experiments and in field trials. Uniquely, the surviving male mosquitoes ensured that the residual activity of released mosquitoes was multigenerational but without long-term persistence of the transgenes in the field.

Uptake of some analogous approaches (including Oxitec first generation mosquitoes, OX513A, Wolbachia-based IIT, irradiation-IIT and classical SIT) has typically been constrained by high production costs and the need for complex and expensive sex-sorting equipment to ensure biting female mosquitoes are not released (Gorman et al., 2016; Zhang et al., 2017; Crawford et al., 2020). Red-eye genetic sexing strains have also been developed for *Aedes aegypti*, but these similarly rely on expensive automated sex-sorting for efficient production (Koskinioti et al., 2021). For some, imperfect manual sex-sorting leads to the need for additional irradiation sterilisation steps (Zheng et al., 2019; Martín-Park et al., 2022), or RNAi-based sterilization (De Castro Poncio et al., 2021) which also add cost and complexity. A further operational restriction is the short shelf-life of adult male mosquitoes (sometimes requiring release within as little as 24 h after production (Crawford et al., 2020)) that has limited the locations in which these tools can be used, as they either require local production facilities, or fast and efficient shipping of live mosquitoes in climate-controlled conditions to release sites. This is normally required as deployment, performed by trained personnel, involves daily or multiple applications per week to achieve and maintain effective overflooding of the target pest population.

By contrast, a genetic sexing mechanism that provides for built-in male-only production, such as in the OX5034 strain, opens new possibilities for effective deployment of male mosquitoes. The self-limiting gene in OX5034 has been shown to be fully penetrant, producing only male OX5034 mosquitoes in the absence of tetracyclines, with very little male fitness cost associated with the transgenes, as demonstrated in laboratory comparisons with WT males and by very effective field suppression of wild *Aedes aegypti* populations. Further, despite the lower female eclosion rates (Table 1) in homozygous OX5034 females when reared on doxycycline, this has not hampered commercial-scale rearing of OX5034. Thereby, OX5034 eggs produced in a central factory location can be shipped internationally (subject to regulatory approvals in any given country), where they can be placed in simple ‘egg-based’ mosquito release devices. Egg-based mosquito release devices have been developed and used successfully to culture and release *Aedes aegypti* during Wolbachia-mediated population replacement programmes for *Aedes aegypti* in Australia (O’Neill et al., 2018) and by Oxitec Ltd. in Brazil for commercial release of the OX5034 strain. Such release devices, containing eggs and larval diet, and requiring only the addition of water, could allow the production of OX5034 mosquitoes in the field, without the need for expensive local production facilities, sex-sorting equipment, or limitations based on shelf-life of male mosquitoes. *Aedes aegypti* eggs, when stored under the appropriate conditions, have a shelf-life of many months (Kliewer, 1961). Results from trials demonstrating the deployment of egg-based mosquito release devices will be reported in separate publications.

Egg-based mosquito release devices also permit simpler release operations that are more sensitive to the mosquito’s biology. Mosquito release devices could potentially release mosquitoes steadily over a period of days or weeks (dependent on ambient conditions), as opposed to burst releases of adults at the roadside at predetermined times during the day. Operational simplicity of this type could facilitate community-based deployment of mosquitoes, as well as by mosquito control professionals. The genetic sexing element (female-specificity of the self-limiting gene) carried by OX5034 also permits the introgression of desirable background genetic traits into the wild population. OX5034, created in the same genetic background as OX513A, is similarly susceptible to insecticides for mosquito control. This genetic background also does not have any increased vector competence compared with wild mosquitoes from a variety of locations (Evans et al., 2019). Indeed, since the unmodified WT mosquitoes used to create this strain are susceptible to insecticides routinely used for mosquito control, including pyrethroids and organophosphates (Carvalho et al., 2015; Patil et al., 2018), the introduction of OX5034 males to a wild, insecticide-resistant population is predicted to dilute insecticide resistance levels (Alphey et al., 2007) by introgressing susceptible alleles into the vector mosquito population, as previously demonstrated for broad-acre crop pests (Harvey-Samuel et al., 2015). However, the self-limiting genes would disappear from the environment over the course of several generations as demonstrated in the field trials described here.

The OX5034 strain harnesses the benefits of pure male release cohorts due to complete trait penetrance and multi-generational suppression, potentially improving efforts to eliminate *Aedes aegypti* from human-inhabited areas. It has shown that genetic sexing strains hold the potential for a step change in operational simplicity, scalability and cost, together with added benefits such as insecticide resistance reversal. The OX5034 strain has now received full commercial and biosafety approval in Brazil from the national biosafety regulator, CTNBio, allowing releases anywhere in the national territory of Brazil (https://www.in.gov.br/web/dou/-/extrato-de-parecer-tecnico-n-6.946/2020-258262552). OX5034 has also received approvals from the United States Environmental Protection Agency (US-EPA) for field pilots in Florida and Texas (https://www.regulations.gov/document/EPA-HQ-OPP-2019-0274-0470). Moreover, trials in both Brazil and the United States have enjoyed high levels of community support for the release of OX5034 mosquitoes. Many other countries where dengue and other arboviruses are endemic also have the relevant regulatory framework to conduct risk assessments for future releases of transgenic mosquito strains for the control of vector mosquitoes. OX5034 and other similar strains may become important tools in the arsenal for controlling populations of *Aedes aegypti*, with the ultimate aim of reducing or even preventing the diseases transmitted by this pernicious vector.

## Data Availability

The datasets used and/or analysed during the current study are available from the corresponding author on reasonable request.
