# Supplementary material for: New self-sexing Aedes aegypti strain eliminates barriers to scalable and sustainable vector control for governments and communities in dengue-prone environments
Source: Front Bioeng Biotechnol. 2022 Oct 25;10:975786. doi: 10.3389/fbioe.2022.975786 (PMC9650594; doi:10.3389/fbioe.2022.975786)
Supplement: Supplementary file 1 [file DataSheet1.docx]

Supplementary Material

# Changes introduced into *Aeadsx* to achieve sex-specific splicing in OX5034 mosquitoes

Table S1. Changes introduced into LA3491 *dsx* gene (numbering is from sequence 149 from US20090183269 patent) to produce the splicing module *Aeadsx* in OX5034.

| Change | Location | Justification |
| --- | --- | --- |
| 2034delT | female-specific exon 5B | Deliberately introduced to open the reading frame in transcript F2 to achieve female-specific tTAV protein production. |
| 2113_2114insC |  |  |
| 2160T>G |  |  |
| 4051_4389del | Exon 6 | The 3’ end of exon 6 was replaced with Ubiquitin and tTAV coding sequences in OX5034, in-frame only in transcript F2. |

# OX5034 Sequence in the *Aedes aegypti* genome

1 gatggtccct agaaacagct ttccaaagga ttgttgttcc atgactcgat atttccatga

61 gtcgatggtc ccttcaatat cgactcatgg aggtttcact gtaaatgaaa ttgcaagtcc

121 acttttttga cattttaccg tgccgggtgt tctctttttt cattaaCCCT AGAAAGATAG

181 TCTGCGTAAA ATTGACGCAT GCATTCTTGA AATATTGCTC TCTCTTTCTA AATAGCGCGA

241 ATCCGTCGCT GTGCATTTAG GACATCTCAG TCGCCGCTTG GAGCTCCCGT GAGGCGTGCT

301 TGTCAATGCG GTAAGTGTCA CTGATTTTGA ACTATAACGA CCGCGTGAGT CAAAATGACG

361 CATGATTATC TTTTACGTGA CTTTTAAGAT TTAACTCATA CGATAATTAT ATTGTTATTT

421 CATGTTCTAC TTACGTGATA ACTTATTATA TATATATTTT CTTGTTATAG ATATCTACCG

481 GGTGTATCAT AGCGATGCGG CCACTCGAGA AATTGAACTG GCTTTACGAG TAGAATTCTA

541 CGCGTAAAAC ACAATCAAGT ATGAGTCATA AGCTGATGTC ATGTTTTGCA CACGGCTCAT

601 AACCGAACTG GCTTTACGAG TAGAATTCTA CTTGTAACGC ACGATCAGTG GATGATGTCA

661 TTTGTTTTTC AAATCGAGAT GATGTCATGT TTTGCACACG GCTCATAAAC TCGCTTTACG

721 AGTAGAATTC TACGTGTAAC GCACGATCGA TTGATGAGTC ATTTGTTTTG CAATATGATA

781 TCATACAATA TGACTCATTT GTTTTTCAAA ACCGAACTTG ATTTACGGGT AGAATTCTAC

841 TTGTAAAGCA CAATCAAAAA GATGATGTCA TTTGTTTTTC AAAACTGAAC TCGCTTTACG

901 AGTAGAATTC TACGTGTAAA ACACAATCAA GAAATGATGT CATTTGTTAT AAAAATAAAA

961 GCTGATGTCA TGTTTTGCAC ATGGCTCATA ACTAAACTCG CTTTACGGGT AGAATTCTAC

1021 GCGTAAAACA TGATTGATAA TTAAATAATT CATTTGCAAG CTATACGTTA AATCAAACGG

1081 ACGCTCGAGG TTGCACAACA CTATTATCGA TTTGCAGTTC GGGACATAAA TGTTTAAATA

1141 TATCGATGTC TTTGTGATGC GCGCGACATT TTTGTAGGTT ATTGATAAAA TGAACGGATA

1201 CGTTGCCCGA CATTATCATT AAATCCTTGG CGTAGAATTT GTCGGGTCCA TTGTCCGTGT

1261 GCGCTAGCAT GCCCGTAACG GACCTCGTAC TTTTGGCTTC AAAGGTTTTG CGCACAGACA

1321 AAATGTGCCA CACTTGCAGC TCTGCATGTG TGCGCGTTAC CACAAATCCC AACGGCGCAG

1381 TGTACTTGTT GTATGCAAAT AAATCTCGAT AAAGGCGCGG CGCGCGAATG CAGCTGATCA

1441 CGTACGCTCC TCGTGTTCCG TTCAAGGACG GTGTTACCGA CCTCAGATTA ATGTTTATCG

1501 GCCGACTGTT TTCGTATCCG CTCACCAAAC GCGTTTTTGC ATTAACATTG TATGTCGGCG

1561 GATGTTCTAT ATCTAATTTG AATAAATAAA CGATAACCGC GTTGGTTTTA GAGGGCATAA

1621 TAAAAGAAAT ATTGTTATCG TGTTCGCCAT TAGGGCAGTA TAAATTGACG TTCATGTTGG

1681 ATATTGTTTC AGTTGCAAGT TGACACTGGC GGCGACAAGA CAATTCTAAT TGGGGTAAGT

1741 TTTCCCGTTC TTTTCTGGGT TCTTCCCTTT TGCTCATCCT TGCTGCACTA CCTTCAGGTG

1801 CAAGTTGAGA TTCAGGCCAC CATGGGTACC GCTAGAGTCG ATCCCACCCC ACCCAAGAAG

1861 AAGCGCAAAC CGGTCGCCAC CATGGCCTCC TCCGAGAACG TCATCACCGA GTTCATGCGC

1921 TTCAAGGTGC GCATGGAGGG CACCGTGAAC GGCCACGAGT TCGAGATCGA GGGCGAGGGC

1981 GAGGGCCGCC CCTACGAGGG CCACAACACC GTGAAGCTGA AGGTGACCAA GGGCGGCCCC

2041 CTGCCCTTCG CCTGGGACAT CCTGTCCCCC CAGTTCCAGT ACGGCTCCAA GGTGTACGTG

2101 AAGCACCCCG CCGACATCCC CGACTACAAG AAGCTGTCCT TCCCCGAGGG CTTCAAGTGG

2161 GAGCGCGTGA TGAACTTCGA GGACGGCGGC GTGGCGACCG TGACCCAGGA CTCCTCCCTG

2221 CAGGACGGCT GCTTCATCTA CAAGGTGAAG TTCATCGGCG TGAACTTCCC CTCCGACGGC

2281 CCCGTGATGC AGAAGAAGAC CATGGGCTGG GAGGCCTCCA CCGAGCGCCT GTACCCCCGC

2341 GACGGCGTGC TGAAGGGCGA GACCCACAAG GCCCTGAAGC TGAAGGACGG CGGCCACTAC

2401 CTGGTGGAGT TCAAGTCCAT CTACATGGCC AAGAAGCCCG TGCAGCTGCC CGGCTACTAC

2461 TACGTGGACG CCAAGCTGGA CATCACCTCC CACAACGAGG ACTACACCAT CGTGGAGCAG

2521 TACGAGCGCA CCGAGGGCCG CCACCACCTG TTCCTGAGAT CTCGACCCAA GAAAAAGCGG

2581 AAGGTGGAGG ACCCGTAAGA TCCACCGGAT CTAGATAACT GATCATAATC AGCCATACCA

2641 CATTTGTAGA GGTTTTACTT GCTTTAAAAA ACCTCCCACA CCTCCCCCTG AACCTGAAAC

2701 ATAAAATGAA TGCAATTGTT GTTGTTAACT TGTTTATTGC AGCTTATAAT GGTTACAAAT

2761 AAAGCAATAG CATCACAAAT TTCACAAATA AAGCATTTTT TTCACTGCAT TCTAGTTGTG

2821 GTTTGTCCAA ACTCATCAAT GTATCTTATC ATGTCTGGAC CTAGGTAAGA GGCGCGCCTA

2881 **CCCACCGTAC TCGTCAATTC CAAGGGCATC GGTAAACATC TGCTCAAACT CGAAGTCGGC**

2941 **CATATCCAGA GCGCCGTAGG GGGCGGAGTC GTGGGGGGTA AATCCCGGAC CCGGGGAATC**

3001 **CCCGTCCCCC AACATGTCCA GATCGAAATC GTCTAGCGCG TCGGCATGCG CCATCGCCAC**

3061 **GTCCTCGCCG TCTAAGTGGA GCTCGTCCCC CAGGCTGACA TCGGTCGGGG GGGCCGTCGA**

3121 **CAGTCTGCGC GTGTGTCCCG CGGGGAGAAA GGACAGGCGC GGAGCCGCCA GCCCCGCCTC**

3181 **TTCGGGGGCG TCGTCGTCCG GGAGATCGAG CAGGCCCTCG ATGGTAGACC CGTAATTGTT**

3241 **TTTCGTACGC GCGCGGCTGT ACGCGGGGCC CGAGCCCGAC TCGCATTTCA GTTGCTTTTC**

3301 **CAATCCGCAG ATAATCAGCT CCAAGCCGAA CAGGAATGCC GGCTCGGCTC CTTGATGATC**

3361 **GAACAGCTCG ATTGCCTGAC GCAGCAGTGG GGGCATCGAA TCGGTTGTTG GGGTCTCGCG**

3421 **CTCCTCTTTT GCGACTTGAT GCTCTTGGTC CTCCAGCACG CAGCCCAGGG TAAAGTGACC**

3481 **GACGGCGCTC AGAGCGTAGA GAGCATTTTC CAGGCTGAAG CCTTGCTGGC ACAGGAACGC**

3541 **GAGCTGGTTC TCCAGTGTCT CGTATTGCTT TTCGGTCGGG CGCGTGCCGA GATGGACTTT**

3601 **GGCACCGTCT CGGTGGGACA GCAGAGCGCA GCGGAACGAC TTGGCGTTAT TGCGGAGGAA**

3661 **GTCCTGCCAG GACTCGCCTT CCAACGGGCA AAAATGCGTG TGGTGGCGGT CGAGCATCTC**

3721 **GATGGCCAGG GCATCCAGCA GCGCCCGCTT ATTCTTCACG TGCCAGTAGA GGGTGGGCTG**

3781 **CTCCACGCCC AGCTTCTGCG CCAACTTGCG GGTCGTCAGT CCCTCAATGC CAACTTCGTT**

3841 **CAACAGCTCC AACGCGGAGT TGATGACTTT GGACTTATCC AGGCGGCTGA** **C***ACCACCGCG*

3901 *CAGGCGCAGC ACCAGGTGCA GGGTGCTCTC CTTCTGGATG TTGTAGTCGC TCAGGGTGCG*

3961 *GCCATCCTCC AGCTGGCGTC CGGCGAAGAT CAGGCGCTGC TGATCCGGCG GGATGCCCTC*

4021 *CTTGTCCTGG ATCTTGGCCT TCACGTTCTC GATGGTATCG CTCGGCTCCA CCTCCAGGGT*

4081 *GATGGTCTTG CCGGTCAGGG TCTTGACGAA* *GATCTG*TCCG GCGAGCTGGA CTGTGACCGG

4141 GTACGAAAAT CGGCACATGG CGACCGTGAC GCAGACGGAG TGCGGGTGAC GGAGATGTTC

4201 TCCTCGTCCG GCCAGTGTCT GTGTCTGCTG GAACGCTTCG GAAAGTAGAT GCAACAAATG

4261 TCGTATC*CTG TTTGAGGGAT TGAATTATTG GGGGGCGAAA GTGGGGAAAG AAAATGTAAA*

4321 *ATAGAACAAA TTATTTATTG TTTATAGAAA AGTGTAATGC TGTTAAGAGG TTAGTATACT*

4381 *CAATGTCTTT CCATCAAATA CGGTTAGAAA TTGTTCATTC TATGGAGATT GCACGACGCC*

4441 *ACGAGTTTGA TTTTACCTTT TGATGTTTGT CACAAACAGC ACTAGGTACT GTAATTTTGA*

4501 *GGTGATGCAA ATAAATTTTG AACCATCCTG CTGAACACAA ATTTATTAGA CGTATTTCGA*

4561 *CGTTTGGTGA GCTCGTTTCC ATACTTGCCT ATCTTACGTT ACTTCAATGA ACAACTAAAT*

4621 *ACACATTTGT ATGCGCTTAT CCCCTAAAGA CGGGTGATAA TGTCAACTGT TTAGCTAATT*

4681 *TCCAAAACAA TCAAACCTTG ATACGAGATA ACTGACTTTG CATCTCTCAA ACTACGATTA*

4741 *ATAGAAAGCT ATAGCAAATT ATGTACTTAG ATAGCATTGG AAAGATGACG CTTTCCGCTA*

4801 *CCTCATAACG CCATTGGCTT TATCTATATG ACTGTTCATA GCTGGAGAGA TGGATTTGAA*

4861 *GGTCTAATTC TAAGTTATCG CATATCAAGG TATCGTTGTG CTGGAAAATC TGATCTGACA*

4921 *GTATCGAAAT AGCGCAACTC TTTGTACCCA ATAATGGAAA GTTCTGATTT ATTGTATTTA*

4981 *TAAAAACGTT GTCTATTTGT TCCGATTTCA GGTCGTCAAA TCACTTGCTA GTAAATAACG*

5041 *TCTCCATAGC AATTATCATT ATTATATACT AAATGAAACG AGCTCACCAA TTAGATAGTT*

5101 *TCAAACAGTT ATACAGTTGC TTCAAACAAC ATACATACAT GCCTTGATAA GTACCGTGCG*

5161 *CCAAATCGAG CTCGCAACAT GAGTATGAAA AGCCACTAGT AACACATTGA TAATCAGCAT*

5221 *AGAATTTAAA ATAAAATAAT ATTTTATACT GTGGATATCT TCATAACACT GCACGCTGAT*

5281 *ATAAAATTCA GAACTACAAA AGGATCGTTG AAATTTTACG TAACTCAACA GAGTAAGGGA*

5341 *GGGGGTCTTC CGAAATGCTA CGGTCTATAC ACAAAATTTA AATTTTTCAC ACAAAAGCCG*

5401 *TTACGTGGAG GAGGGAGGGG TTCTAAAATT GGCAAACTTT GCGTCACGTA ATATTTGAAT*

5461 *CATCCCAAAG AAAATAAACT TCTATGCTGA TTATAACGCG CGCAGAACAA TGGTTGCGGT*

5521 *GAGAGACATT AACAGGCTTG TTTGTGGTGC TGAAATAGAA CTTGTCATCA ATATGTTTTA*

5581 *GCTGGTTAAA CTATACAATC ATTACGTAGC CTGAAAATCA CCCTTGAAAA GGCCGAATAT*

5641 *ATGACGAAAA GACACACTCT CCAACTCAAA AGGCAAACTC AACGTGGTCG TGCACAACCT*

5701 *CCAATAGCAG TAC*CTGTCGG AGCCGTTTGG CAACGGCCAG CTACCAAAAT ACGCTCGCAA

5761 TGGCATGCAA GCTACAGAGA GATAAGTGTT TATCAGATCA TTTTGGGCAC CGAAACCGAC

5821 CGATGTCGGA AACGATTGAA GAGATATAAT CTCTGGTTTG TAGATTGTAG GATGGTTGGT

5881 TGAAGATCCG GTTTCCCGGA TTTTTTCGGA TGGATGTTGC TTGTTGATGA TTCTGCTGTC

5941 GTCGTTTTTT TCCGGTGGCA GATGGAACAG CCTCACTTCG GCTTTCGAAA CACAATCTTC

6001 AAAGTTAAGT ACTACTGCTG TTTTGCATTT TTTAAATTTT CCCTCTGAAA CTTGCTTTGC

6061 ACTATTGGTT TCATCACTTG TTTGCACTTT CACACTCTTT AGGAACGCTG TCTCACAAGT

6121 AGAGCTCACG TGGTAGCCCC AGAATGGCTG CATGTCGCTA TTATCGTTAA ACAGCATTTG

6181 CA*CTGTGGTC ATTATGTTGT TTGTATTAGA GTTCGTCGCG TTCGTGGAAT TGGGAAGGAG*

6241 *AAGATACAGA ATTACTCAAA TGAAACCATT CCACGGGAGA ATACATTTCA GGTTTAATCT*

6301 *TATTCTTCTA GGACGAAAGC CCATCGACAG AGTCTTGCAG GCTTCCGTCG ATCGACTTTC*

6361 *ACCCGTGGAT GCTAAGGAAG CTTATAATGA C*CTCAACATT CTCCGCGCAC AGGTTGGCTC

6421 TATTCTGTTT CACAGTTTCC GGTGCAGTTG TGACGAACTC AGACGAATAC CCACGATTGT

6481 ATGTCCAACC TCATTTTTTA TCTTTGTAAA CTAACGTCGA AAAATCTAGA TACTACATTT

6541 CTGCTTTGCT TCATCTTACA CTAATCACTA GTTTGAACTT GCGGGTTTTC CGTTATGCTT

6601 TGTAAATATG CGATGCTTTA GAGTTTTCTT CGTTCCGATT CTTCTTTGCA TTCGATTGCT

6661 TCTTCCGTCG AATCGATCTG ATCTTCGTGG TTTATTCTTG TTTCGGTTCG ACCTTTGCCG

6721 CAGCGCAGTG GGTCGTGCTG ATCGTGTAAA AAGTCTATCA TCCGGACTGG CGCGTCGTAC

6781 TGCGCAACTC TACACCGTCG AACATGTTCA GATTGTGCAA TCGTGAGTAT TCATTGACCA

6841 CGGCTTGAC*C TGCGAGGCAG AGAAGAACAG TTGGATTTTT CGGATATTGG TACGACCCGG*

6901 *GGGCCGCGTT GTCATCAGTT GCATGAATCG TTGGTCCAAG TTCGACGAAA CGATATGGAC*

6961 *ATCGGTGTTT CGGTGGACCA AGATCGACGA CACGATCTTG GTCATGAGTG TTTTTCGGTG*

7021 *GACCTAGAGA TATTGCAACG ACCGGAGTGG AATACGACGG TACGATGTTG GTTTGTACTG*

7081 *TTCTGGACGC TAGTTACTTC ATTGATACGA TAAAGTTTAC ATTCGTCATA TCTCTTGCTT*

7141 *TTCTTGAATC CAAATGCTTA GGACGTGTAA CCTTCACAAA CCGTCATAAA TCAGTTTCGA*

7201 *TTCACTACAT GTTGTAGTTA TTCAGGCTCT TATAATTGAA TATTCAAAAA TCGAATTTTC*

7261 *TATTTTATCT TGATCAGAGG ATATAACTCG CTTAAAATGC ACAATTTTAT TAGCGACACC*

7321 *ATGTGGATTT GTTTTAATTG AAACCTCTAT CTTCTCATAT GTATCACGAT ATAAAATGCT*

7381 *CATTTTATTG ACTGTTTAAC GATAAACTTG CGACGATCGA CGCACCACCG ACCTAATTCC*

7441 *ATTGTGGAAG CAAGGGGCAC TGCAATACCG AAATGTGAAG TAAATTTCAA ATCTGCTATT*

7501 *ATAGACGATG ATCTAATACT CTTGAATGGT CTTAAACGTG AGTTGTATTT CAAGAAGTTA*

7561 *TGACGATTCG ATTTTGGGGC CATTATGACC CCAAAACCCA GCCAACGTAA CTTTTATTAG*

7621 *TACAGACAGA AGGTCAAGCG TGCAAGTCTT TCATCCGTGT GTCAATAAGG CCATCAGTTG*

7681 *AAACCGTGTC AATTAACCCT CCAGTTAACC CTTTTAACTT TTACCAGGAC AAACCAATGA*

7741 *CTTCGTGCGC AAATTCCACC ACTCGTTGTC TCAGGCCTTG AGTTGTTGTT TGATAAGAAT*

7801 *GGGGGATGTC AAGTCGGGGA GCGTAGCCCA ACAGGCTACG GAAACTGCAT GATGGCAGTG*

7861 *TTTGATCCAG GGCACTGTTG GGAATAGACT CCGTCGACCG AAGATCCCAG ATGTCCTGAA*

7921 *ACTCAATAAT AAGCGTTAGC AGTTACAAAA TGGGAGCACC CAGGAAGTGA GTGACACCCG*

7981 *ATCGATACCT CGGAAACAGT CCCAACCGGT AAGAACCCCC ATAC*CTTCGT CAATCCGTTG

8041 GCGCGCTTTA TTGACGTCTC CGTCGGCGCC TTTCAGTATC ACGTACATCA GGGGCATCAT

8101 CTCCCAGGGG TATCGCAGCT TCTCCAGGAG CCATTGAGAT CGTTTGACAA GTTCGTCGTC

8161 CATGGTGGCA GATTGTTTAG CTTGTTCAGC TGCGCTTGTT TATTTGCTTA GCTTTCGCTT

8221 AGCGACGTGT TCACTTTGCT TGTTTGAATT GAATTGTCGC TCCGTAGACG AAGCGCCTCT

8281 ATTTATACTC CGGCGCTCGT TTTCGAGTTT ACCACTCCCT ATCAGTGATA GAGAAAAGTG

8341 AAAGTCGAGT TTACCACTCC CTATCAGTGA TAGAGAAAAG TGAAAGTCGA GTTTACCACT

8401 CCCTATCAGT GATAGAGAAA AGTGAAAGTC GAGTTTACCA CTCCCTATCA GTGATAGAGA

8461 AAAGTGAAAG TCGAGTTTAC CACTCCCTAT CAGTGATAGA GAAAAGTGAA AGTCGAGTTT

8521 ACCACTCCCT ATCAGTGATA GAGAAAAGTG AAAGTCGAGT TTACCACTCC CTATCAGTGA

8581 TAGAGAAAAG TGAAAGTCGA AACCTGGCTA GCAGATCTCA TATGATTAAG AGGCGCGGTA

8641 AACCGCAAAT GGTTATGTAT TATAATCAAA CTAAAGGCGG AGTGGACACG CTAGACCAAA

8701 TGTGTTCTGT GATGACCTGC AGTAGGAAGA CGAATAGGTG GCCTATGGCA TTATTGTACG

8761 GAATGATAAA CATTGCCTGC ATAAATTCTT TTATTATATA CAGCCATAAT GTCAGTAGCA

8821 AGGGAGAAAA GGTTCAAAGT CGCAAAAAAT TTATGAGAAA CCTTTACATG AGCCTGACGT

8881 CATCGTTTAT GCGTAAGCGT TTAGAAGCTC CTACTTTGAA GAGATATTTG CGCGATAATA

8941 TCTCTAATAT TTTGCCAAAT GAAGTGCCTG GTACATCAGA TGACAGTACT GAAGAGCCAG

9001 TAATGAAAAA ACGTACTTAC TGTACTTACT GCCCCTCTAA AATAAGGCGA AAGGCAAATG

9061 CATCGTGCAA AAAATGCAAA AAAGTTATTT GTCGAGAGCA TAATATTGAT ATGTGCCAAA

9121 GTTGTTTCTG ACTGACTAAT AAGTATAATT TGTTTCTATT ATGTATAAGT TAAGCTAATT

9181 ACTTATTTTA TAATACAACA TGACTGTTTT TAAAGTACAA AATAAGTTTA TTTTTGTAAA

9241 AGAGAGAATG TTTAAAAGTT TTGTTACTTT ATAGAAGAAA TTTTGAGTTT TTGTTTTTTT

9301 TTAATAAATA AATAAACATA AATAAATTGT TTGTTGAATT TATTATTAGT ATGTAAGTGT

9361 AAATATAATA AAACTTAATA TCTATTCAAA TTAATAAATA AACCTCGATA TACAGACCGA

9421 TAAAACACAT GCGTCAATTT TACGCATGAT TATCTTTAAC GTACGTCACA ATATGATTAT

9481 CTTTCTAGGG ttaagatgtg tttattcttt ctaaatacag catctttgaa acatctaaca

9541 agtctacttg agtattccat tcattgaata acaatgcaac gcattttgat aacttttcaa

9601 accagcaact gtgtttcgtg cgcagcaaca g

**Figure S1. The sequence of the OX5034 rDNA insertion obtained by Sanger sequencing of overlapping PCR fragments. The rDNA sequence is in capital letters and that of the flanking genomic DNA in small letters. The TTAA sequences (characteristic of *piggyBac*-based insertion sites) are underlined. The sequence of DsRed2-OX5034 is highlighted and underlined. The sequence of tTAV-OX5034 is indicated in bold and highlighted. The sequence of ubiquitin is in *italics and highlighted.* The sequence of the *Aeadsx* splicing module is also annotated, with exons highlighted and introns in *italics*.**

# Insecticide susceptibility data for OX5034 mosquitoes

OX5034 mosquitoes, and the WT strain used to generate OX5034, were tested for susceptibility to several commonly used insecticides. Testing was carried out by the Liverpool Insect Testing Establishment (LITE). Four replicates of 25 mosquitoes (2-5 day old adult females) of each strain were exposed for one hour to standard WHO-made filter papers carrying discriminating doses of Permethrin, Deltamethrin, Propoxur and Malathion. Mortality was scored at 60 minutes and again at 24 h after the start of exposure. Complete susceptibility of both strains was demonstrated against all insecticides except for Propoxur, which killed 94% and 89% of WT and OX5034 mosquitoes, respectively (Table S2).

Table S2. Results of adulticide susceptibility bioassays, shown as percentage of 2-5 day old female adults that were knocked down immediately post-exposure (60 minutes) or killed by 24 hours post-exposure to standard WHO papers treated with discriminating doses of insecticide.

| **Strain** | **Insecticide** | **Knockdown (60 minutes)** | **Mortality (24 hours)** |
| --- | --- | --- | --- |
| **Wild-type *Aedes aegypti* (from Chiapas, Mexico)** | Control (no insecticide) | 0% (n=75) | 0% (n=75) |
|  | 0.75% permethrin | 100% (n=100) | 100% (n=100) |
|  | 0.05% deltamethrin | 100% (n=100) | 100% (n=100) |
|  | 0.1% propoxur | 92% (n=104) | 94% (n=104) |
|  | 5% malathion | 100% (n=100) | 100% (n=100) |
| **OX5034 *Aedes aegypti*** | Control (no insecticide) | 100% (n=100) | 100% (n=175) |
|  | 0.75% permethrin | 94% (n=94) | 100% (n=94) |
|  | 0.05% deltamethrin | 98% (n=100) | 100% (n=100) |
|  | 0.1% propoxur | 89% (n=101) | 89% (n=101) |
|  | 5% malathion | 100% (n=91) | 100% (n=91) |

OX5034 and the comparator WT strain were also assessed for susceptibility to the larvicide temephos. For each temephos concentration (Table S3), four replicates of approximately 25 larvae each (L3-L4) were set up. Each replicate used 200 mL water containing either the specified temephos concentration (stock solutions in acetone), or the same volume of acetone for the negative controls. A clean Pasteur pipette was used to transfer the mosquito larvae into each exposure or control pot, noting this as the start time on the recording sheet. Pots were left for 24 hours in the insectary at standard conditions. At the end of the exposure period, a set of weigh boats were labelled with dose and strain details and used as recovery pots for larvae. 150 mL deionized water was added to each weigh boat. Mosquito larvae were transferred to the new weigh boats for a minimum of 1 hour to allow them to recover (if insufficiently affected by the larvicide). Mortality of the larvae was recorded at the end of the 1-hour recovery period. Larvae were considered dead if they did not display swimming movement when gently prodded.

Results of the larvicide susceptibility assay are presented in Table S3, presented as percentage mortality (with n values) for each test, scored 24 h after larval exposure, compared to the negative control.

Mortality in negative control replicates averaged less than 5% for each treatment (averages of 4 treatments shown in Table S3), so no Abbott’s correction was applied. Some pupation was seen in most treatments (except 1mg/L and 0.1 mg/L), and these individuals were excluded from the calculations.^[[1]](#footnote-1)^ In no case did more than 10% of the control larvae pupate in the course of the experiment.

OX5034 was completely susceptible to temephos at 1mg/L, 0.1mg/L and also at the proposed WHO discriminating dose of 0.012 mg/L.

The WT strain was completely susceptible to temephos at 1 mg/L and 0.1 mg/L but 3% survival was observed at the discriminating dose of 0.012 mg/L (97% mortality).

Table S3. Results of larvicide susceptibility bioassays. For each dose, the percentage of larval mortality is given as an average of the four replicates. The WHO discriminating dose is shown in bold.

| *Aedes aegypti* strain | 1 mg/L  (% mortality) | 0.1 mg/L  (% mortality) | **0.012 mg/L**  **(% mortality)** | 0.003 mg/L  (% mortality) | 0.001 mg/L  (% mortality) | 0.0003 mg/L  (% mortality) | Acetone-only control  (% mortality) |
| --- | --- | --- | --- | --- | --- | --- | --- |
| OX5034 | 100 | 100 | **100** | 23.7 | 2.0 | 2.0 | 0 |
| Latin wild-type | 100 | 100 | **96.9** | 8.0 | 4.0 | 1.0 | 1.0 |

Table S4. LC_50_ and LC_95_ for each strain, calculated from dose-response curves plotted from mortality at each dose of temephos.

|  | LC_50_ (temephos) | LC_50_ 95% CI |
| --- | --- | --- |
| OX5034 | 0.0039 | 0.0030 – 0.0047 |
| Latin WT | 0.0055 | 0.0047 – 0.0064 |

1. WHO guidelines indicate that “*if more than 10% of the control larvae pupate in the course of the experiment, the test should be discarded and repeated*.” ^61^ [↑](#footnote-ref-1)
